# *De novo* assembly of the zucchini genome reveals a whole‐genome duplication associated with the origin of the *Cucurbita* genus

**DOI:** 10.1111/pbi.12860

**Published:** 2017-12-04

**Authors:** Javier Montero‐Pau, José Blanca, Aureliano Bombarely, Peio Ziarsolo, Cristina Esteras, Carlos Martí‐Gómez, María Ferriol, Pedro Gómez, Manuel Jamilena, Lukas Mueller, Belén Picó, Joaquín Cañizares

**Affiliations:** ^1^ Institute for the Conservation and Breeding of Agricultural Biodiversity (COMAV‐UPV) Universitat Politècnica de València Valencia Spain; ^2^ Department of Horticulture Virginia Polytechnic Institute and State University Blacksburg VA USA; ^3^ Instituto Agroforestal Mediterráneo (IAM) Universitat Politècnica de València Valencia Spain; ^4^ IFAPA Centro La Mojonera La Mojonera Almería Spain; ^5^ Department of Biology and Geology Research Centers CIAIMBITAL and CeiA3 University of Almeria Almería Spain; ^6^ Boyce Thompson Institute for Plant Research Ithaca NY USA

**Keywords:** genome, crop, zucchini, whole‐genome duplication, Cucurbitaceae, transcriptome

## Abstract

The *Cucurbita* genus (squashes, pumpkins and gourds) includes important domesticated species such as *C. pepo, C. maxima* and *C. moschata*. In this study, we present a high‐quality draft of the zucchini (*C. pepo*) genome. The assembly has a size of 263 Mb, a scaffold N50 of 1.8 Mb and 34 240 gene models. It includes 92% of the conserved BUSCO core gene set, and it is estimated to cover 93.0% of the genome. The genome is organized in 20 pseudomolecules that represent 81.4% of the assembly, and it is integrated with a genetic map of 7718 SNPs. Despite the small genome size, three independent lines of evidence support that the *C. pepo* genome is the result of a whole‐genome duplication: the topology of the gene family phylogenies, the karyotype organization and the distribution of 4DTv distances. Additionally, 40 transcriptomes of 12 species of the genus were assembled and analysed together with all the other published genomes of the Cucurbitaceae family. The duplication was detected in all the *Cucurbita* species analysed, including *C. maxima* and *C. moschata*, but not in the more distant cucurbits belonging to the *Cucumis* and *Citrullus* genera, and it is likely to have occurred 30 ± 4 Mya in the ancestral species that gave rise to the genus.

## Introduction

*Cucurbita pepo* L. is the main crop of the *Cucurbita* genus. At the subspecies level, three taxa are recognized as follows: subsp. *pepo*, known only in cultivation (zucchini, pumpkins and summer and winter squashes), subsp. *ovifera* (L.) Decker (=subsp. *texana* (Scheele) Filov), known in cultivation and in the wild (scallop and acorn squashes, ornamental gourds), and subsp. *fraterna* (L. H. Bailey) Lira, Andres & Nee (=*C. fraterna* L. H. Bailey), known only in wild populations (Andres, 1987; Decker‐Walters, 1990; Paris *et al*., 2015). Subspecies *pepo* and *ovifera* include many edible‐fruited cultivar groups, such as pumpkin, vegetable marrow, cocozelle, zucchini, acorn, scallop, straightneck and crookneck. There is evidence of an early domestication of this species (Smith, 1997), with more than one domestication event in Mexico and the United States (Smith, 2006), and it has had two different diversification processes: one in America and one in Europe (Paris *et al*., 2012), where zucchini and other elongated forms, such as vegetable marrow and cocozelle, were developed.

*Cucurbita pepo* is an economically important crop. Its production reached 25 million tons in 2014, with nearly two million cultivated hectares (http://www.fao.org/faostat/en). Cultivated varieties display a rich diversity of vine, flowering and fruit traits, and among them, cultivars of the zucchini group rank among the highest‐valued vegetables worldwide (Formisano *et al*., 2012). The *Cucurbita* genus and the Cucurbitaceae family contain other important crops, such as other squashes, pumpkins and gourds (*Cucurbita maxima* Duchesne and *Cucurbita moschata* (Duchesne ex Lam.) Duchesne ex Poir.), melon (*Cucumis melo* L.), cucumber (*Cucumis sativus* L.) and watermelon (*Citrullus lanatus* (Thunb.) Mansf).

Despite the agronomic importance of the species, prior to the genome assembly presented here, few *C. pepo* genetic and genomic resources were available: a first generation of genetic maps constructed with AFLP, RAPD and SSR markers (Brown and Myers, 2002; Gong *et al*., 2008; Lee *et al*., 1995; Zraidi and Lelley, 2004, 2007) that were later improved with SNPs (Esteras *et al*., 2012) and several transcriptomes (Blanca *et al*., 2011; Vitiello *et al*., 2016; Wyatt *et al*., 2015; Xanthopoulou *et al*., 2016). More recently, a high‐density SNP‐based genetic map was developed using a RIL population derived from the cross between two *C. pepo* subspecies (subsp. *pepo* Zucchini × subsp. *ovifera* Scallop) (Montero‐Pau *et al*., 2017). This map was developed to assist us with the *de novo* assembly process.

In this study, we present a *de novo* assembly of the *C. pepo* genome, a high coverage transcriptome of *C. pepo* and 40 transcriptomes of 12 species of the *Cucurbita* genus. Comparative and phylogenetic analyses show that a whole‐genome duplication (WGD) occurred just before the speciation events that created this genus. All these resources and several previous transcriptome and draft genome versions are publicly available at https://bioinf.comav.upv.es/downloads/zucchini.

## Results

### De novo genome assembly

The complete genome of *Cucurbita pepo* has been sequenced using a whole‐genome shotgun sequencing approach. The zucchini type (*C. pepo* subsp. *pepo*) accession MU‐CU‐16 was selfed four times before sequencing. This accession is characterized by early flowering, a bushy growth habit, high production and uniform, cylindrical, dark‐green fruits. This accession was also used as parental in two previous genetic maps (Esteras *et al*., 2012; Montero‐Pau *et al*., 2017). One paired‐end library, with an insert size of 500 bp, and four mate‐pair libraries, with sizes of 3, 7, 10 and 20 Kb, were created and sequenced in five Illumina Hiseq2000 lanes, resulting in a genome coverage of 254× for the pair‐end library and 54, 46, 65 and 62 X for the 3‐, 7‐, 10‐ and 20‐Kb libraries, respectively (Table S2). All reads were quality‐trimmed and filtered. Additionally, approximately 40% of the 3‐ and 7‐Kb mate‐pair reads were found to be chimeric and were filtered out by comparing them against a preliminary assembly (see Table S2, Figure S1). This chimeric filtering doubled the contig N50 and tripled the scaffold N50 of the final assembly. Finally, 503‐M filtered pair‐end reads and 185‐M mate‐pair reads were used in the assembly. The genome was assembled by SOAPdenovo2 (Luo *et al*., 2012). A *k*‐mer size of 41 was chosen for the final assembly because it yielded the highest N50 values (Figure S2). The SOAPdenovo2 scaffolds were broken, and the scaffolding was redone with SSPACE (Boetzer *et al*., 2011) and GapCloser (Luo *et al*., 2012). The final assembly covered 263 Mb in 26 005 scaffolds and 32 754 contigs with a contig N50 of 110 Kb (L50 = 606 contigs) and a scaffold N50 of 1.8 Mb (L50 = 42 scaffolds) (Table 1). Completeness of the *de novo* assembly was assessed with BUSCO using a plant‐specific database of 1440 genes. Of this total, 92.1% were found to be complete (73.1% as single genes and 19.0% as duplicated genes) and 2.1% were found to be fragmented. The Illumina RNAseq reads obtained from the MU‐CU‐16 accession were mapped with HISAT2 with this genome as reference with a 91.9% success rate. The pair‐end reads used to build the assembly were mapped against the assembly with a success rate of 99.4%. From the *k*‐mer distribution, the genome size was estimated to be 283 Mb; thus, 93.0% of the genome would be covered by the assembly. Chloroplastic and mitochondrial scaffolds were detected using Blast: 250 scaffolds were identified as mitochondrial and 13 as chloroplastic (Table S3).

**Table 1:** Assembly statistics of *C. pepo* genome version 4.1

Parameter	Value
GC content (%)	36.52
No. of contigs (≥0 bp)	32 754
No. of contigs (≥500 bp)	13 896
No. of contigs (≥1000 bp)	8217
Bases in contigs (≥0 bp)	247 816 249
Bases in contigs (≥1000 bp)	238 245 128
Largest contigs (bp)	639 487
N50 contig size (bp)	110 136
N75 contig size (bp)	49 377
L50 contig number	606
L75 contig number	1407
No. of scaffolds (≥0 bp)	26 025
No. of scaffolds (≥500 bp)	7994
No. of scaffolds (≥1000 bp)	3709
Bases in scaffolds (≥0 bp)	263 500 453
Bases in scaffolds (≥500 bp)	258 108 973
Bases in scaffolds (≥1000 bp)	255 237 628
Largest contig (bp)	6 123 784
N50 scaffold size (bp)	1 749 822
N75 scaffold size (bp)	453 344
L50 scaffold number	42
L75 scaffold number	112

The genetic map developed with the RIL population of zucchini x scallop (accessions MU‐CU‐16 × UPV‐196) (Montero‐Pau *et al*., 2017) was used to detect chimeric scaffolds and to anchor and order the scaffolds into pseudomolecules. A total of 7718 SNPs (average of 386 markers/linkage group) were located in the map. Based on the relationship of physical and genetic distances and the presence of the same scaffold in more than one linkage group, 22 of the 26 005 scaffolds were identified as chimeric. Those scaffolds were visually inspected and split. In a first attempt, a total of 181 scaffolds could be anchored to 21 pseudomolecules, which represents the 81.4% of the assembled genome. Finally, after the integration of this genetic map with the genetic maps developed by Esteras *et al*. (2012), which were based on data from the F_2_ of the same cross, and the genetic map of Holdsworth *et al*. (2016), all scaffolds were grouped into 20 pseudomolecules (Table 2 and Table S4). Between 4 and 19 scaffolds were anchored to each pseudochromosome with a length between 8.1 Mb and 21.3 Mb (Table 2). Of the remaining 25 344 scaffolds, 3295 were longer than 1 Kb and 365 were longer than 20 Kb. The average correlation between physical distance and genetic distance was 0.98 (0.94–1.00; Figure S3). This assembly constitutes genome version 4.1. Some other previous versions were made available to the *Cucurbita* community, but none were published.

**Table 2:** Pseudochromosome summary. Number of scaffolds anchored to each pseudochromosome, total length, length without the 1000 N spacers and number of genes

Molecule	#Scaffolds	Length (bp)	Length without N spacers (bp)	Number of genes
Cp4.1LG01	19	21 320 769	21 302 769	3258
Cp4.1LG02	16	14 376 414	14 361 414	1981
Cp4.1LG03	12	13 772 414	13 761 414	2178
Cp4.1LG04	5	12 709 140	12 705 140	1870
Cp4.1LG05	8	10 865 678	10 858 678	1753
Cp4.10LG06	11	10 677 745	10 667 745	1407
Cp4.1LG07	14	10 147 556	10 134 556	1404
Cp4.1LG08	4	10 059 303	10 056 303	1503
Cp4.1LG09	10	9 920 322	9 911 322	1608
Cp4.1LG10	8	9 835 092	9 828 092	1432
Cp4.1LG11	11	9 833 969	9 823 969	1319
Cp4.1LG12	5	9 824 194	9 820 194	1388
Cp4.1LG13	8	9 354 089	9 347 089	1400
Cp4.1LG14	5	8 955 933	8 951 933	1263
Cp4.1LG15	4	8 816 444	8 813 444	1136
Cp4.1LG16	10	8 691 934	8 682 934	1114
Cp4.1LG17	9	8 680 504	8 672 504	1281
Cp4.1LG18	7	8 333 454	8 327 454	1186
Cp4.1LG19	8	8 246 682	8 239 682	1386
Cp4.1LG20	7	8 120 804	8 114 804	1000

### Transcriptome and genome annotation

Two cDNA libraries were created for the parent accessions of the RIL population using pooled RNA from different vegetative and reproductive tissues. More than 228 million reads were added to the previously available 454‐based transcriptome (Blanca *et al*., 2011). They were used to create a new transcriptome assembly (version 3.0, available at https://bioinf.comav.upv.es/downloads/zucchini) and to annotate the genome. The transcriptome assembly identified 108 062 transcripts, 65 990 of which included an ORF. GO terms could be assigned to 71.5% of the coding transcripts. Completeness of the *de novo* assembly assessed with BUSCO showed that 91.0% of the 1440 BUSCO groups searched were found to be complete and 4.0% were found to be fragmented.

**Figure 1:**
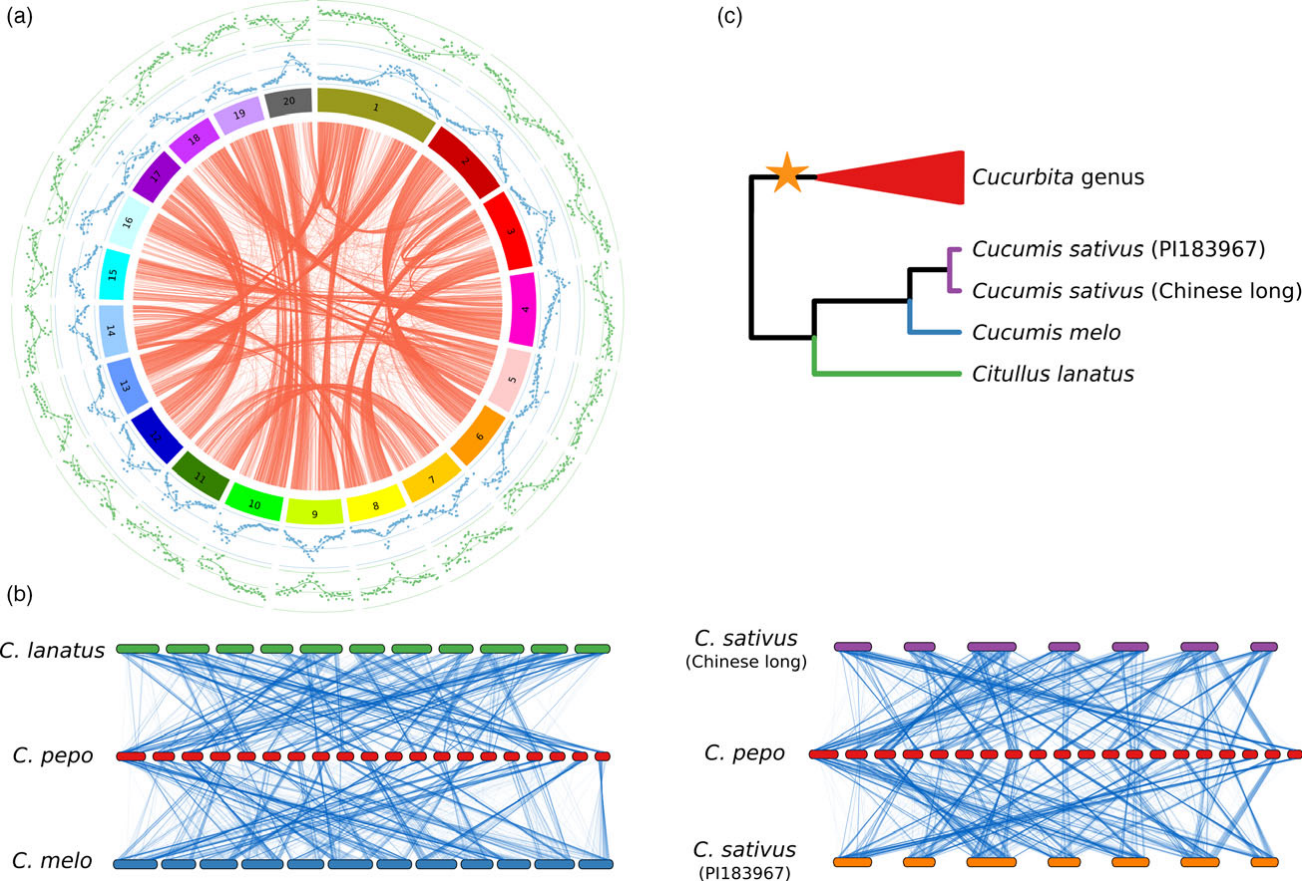
Genome organization. (a) Circos plot showing paralogous gene pairs in *Cucurbita pepo* (red lines). Outer plots represent the proportion of repetitive (blue) or gene‐encoding (green) DNA by 200‐Kb windows. (b) Genomic synteny between *Cucurbita pepo* and *Cucumis melo, Cucumis sativus* and *Citrullus lanatus*. Lines join single‐copy orthologs. (c) Summary diagram with the species phylogeny showing the WGD event.

The genome annotation resulted in 34 240 predicted gene models, of which 27 870 were protein‐coding genes (Table 3). These results are similar to those found in melon and cucumber (Garcia‐Mas *et al*., 2012; Huang *et al*., 2009). The average gene size was 3450 bp with an average of 5.4 exons (Figure S4). The gene models covered 118 Mb, and their coding regions 35 Mb, which represent 45.3% and 13.7% of the assembled genome, respectively, and indicate a high degree of genome compaction (Figure 1a). GO terms could be assigned to 19 784 protein‐coding genes out of 27 870 (71.0%; Figures S5 and S6). Functional descriptions were added to 76.6% of transcripts using AHRD, and 79.2% were tagged with an InterPro protein domain.

**Table 3:** Genome annotation summary

Number of genes	34 240
Number of protein‐coding genes	27 870
Number of exons	184 243
Number of CDSs	166 271
Number of introns	150 003
Number of 5′ UTRs	21 701
Number of 3′ UTRs	22 296
Number of tRNAs	6370
% of gene with introns	70.8
Mean number of exons per gene	5.4
Mean gene length (bp)	34503.4
Mean exon length (bp)	274.9
Mean intron length (bp)	450.0

### Repetitive elements

We identified that 93 Mb (37.8% of the assembly) consisted of repetitive elements (REs) (Figure S7 and Table S5). Long terminal repeats (LTR) represented 50.7% of the identified REs. *Gypsy* and *Copia* were the most abundant LTR superfamilies (24.2% and 19.8% of identified REs and 3.3% and 2.7% of the total genome). The *Gypsy* LTR abundance is similar to that found in *C. melo, C. lanatus* and *C. sativus,* which ranged from 19.5% to 34.4%, whereas the *Copia* family was less represented than in other Cucurbitaceae genomes (30.9%–34.4%). Other two LTR superfamilies were more abundant in the *C. pepo* genome compared to the other cucurbits: *Cassandra* (3% of identified RE vs. 0.1%–0.8%) and *Caulimovirus* (2.1% vs. 0.26%–0.9%). Satellites and simple repeats constituted 25.2% of all identified REs, which is a larger fraction than in related Cucurbitaceae species (4.4%–12.4%). *Copia* and *Gypsy* REs were assigned to their distinct families by building two phylogenetic trees (one for *Copia* and one for *Gypsy*; Figure S7). All *Copia* and *Gypsy* families previously identified in *C. melo, C. lanatus* and *C. sativus* were also present in *C. pepo,* except for *Copia/Bianca* and *Gypsy/Ogre* families. In these trees, the Gypsy/*Galadriel* and *Copia/Tork4* families were overrepresented in *C. pepo*, so they seem to have undergone a diversification process in this species. Finally, approximately, 24% of REs were not assigned to any class of repetitive or transposable elements (TEs).

### Comparative genomics

**Figure 2:**
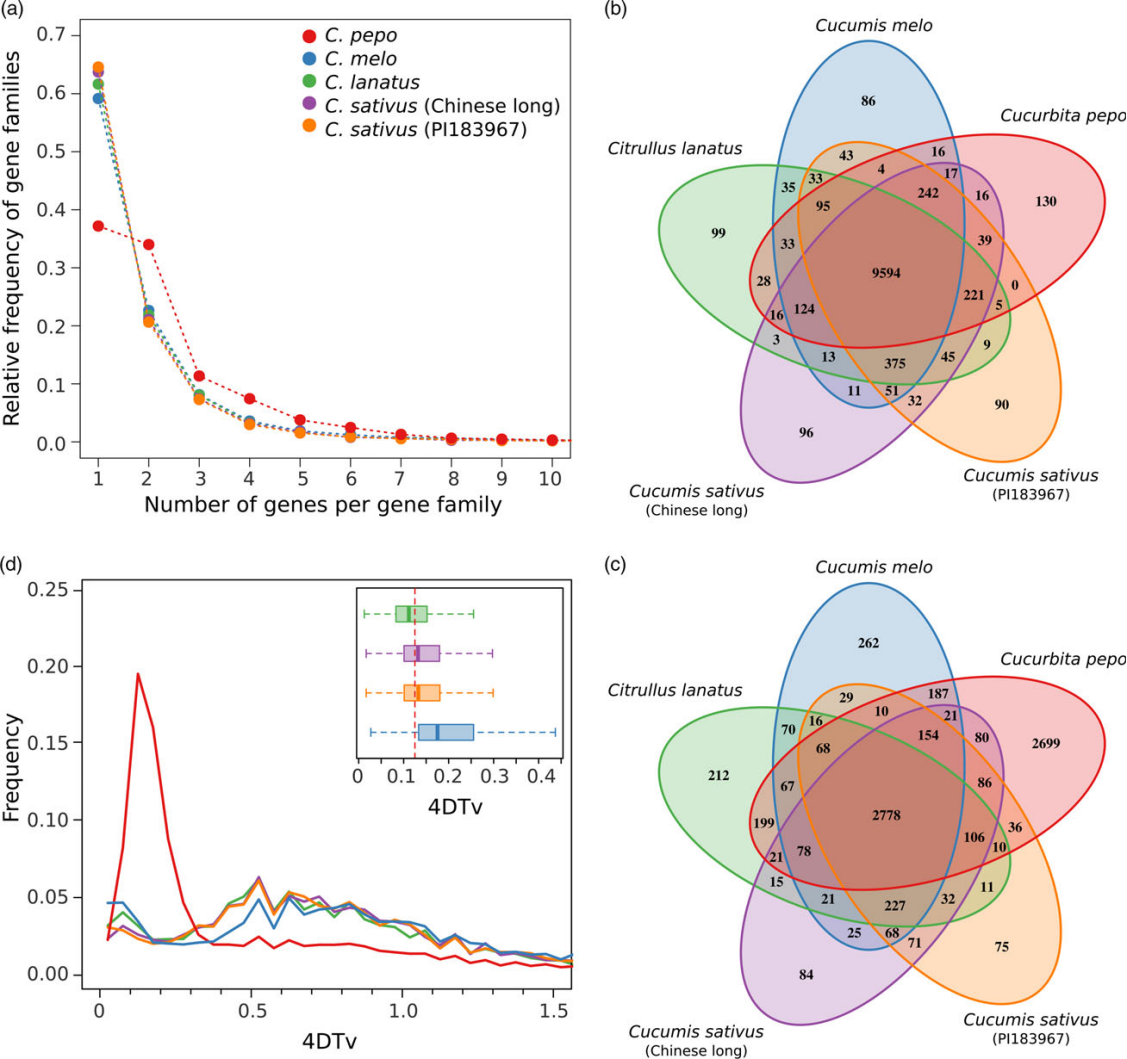
Genome duplication. (a) Distribution of the number of gene families based on the number of gene copies for *Cucurbita pepo, Cucumis melo, Cucumis sativus* and *Citrullus lanatus*; (b) Venn diagram showing the number of gene families, and (c) the number of duplicated gene families shared among the cucurbit genomes; (d) distribution of the rate of transversions on fourfold degenerate synonymous sites (4DTv) among paralogs for the five studied genomes. The inset shows the boxplots for the 4DTv distribution between ortholog copies of *C. pepo* and the rest of cucurbit species. The red dashed line shows the duplication event in *C. pepo*.

Genes, represented by the longest protein of the four cucurbit crops (*Cucurbita pepo*,* Cucumis melo, Citrullus lanatus* and two *Cucumis sativus* cultivars var. *sativus,* Chinese long; and var. *hardiwickii,* PI 183967), were grouped into gene families using OrthoMCL. The percentage of genes that could be assigned to a gene family in these species ranged from 91.2% to 72.8% (Table S6). In *C. pepo*, the number of gene families with two or more paralogs was higher than in the other crops (Figure 2a). Most *C. pepo* gene families were also present in the other cucurbits (Figure 2b); however, many of them had more than one gene in *C. pepo* (Figure 2c). Most of the zucchini paralogs were organized in large syntenic regions that covered most of the genome (Figure 1). Synteny with the other cucurbit species showed that, despite some conserved synteny, an extensive chromosomal rearrangement has occurred (Figure 1). The high number of paralogous genes detected and their synteny suggests that *C. pepo* could have undergone a WGD (Tables S7, S8 and S9).

The rate of transversions on fourfold degenerate synonymous sites (4DTv) is a neutral genetic distance that can be used to estimate relative timing of evolutionary events. The distribution of 4DTvs among paralog pairs for all species other than *C. pepo* showed a wide peak that ranged from 0.4 to 1.1 with a maximum approximately 0.6 (Figure 2d), whereas for *C. pepo,* a more recent and narrower peak centred approximately 0.12 was found. The relative date of speciation can also be predicted by computing the 4DTvs between orthologous genes of any pair of species. These analyses showed that the speciation event that gave rise to the *Cucurbita* genus, represented by the pairwise 4DTv distributions of *C. pepo* against *C. lanatus, C. sativus* and *C. melo,* occurred almost simultaneously with the duplication event found in *C. pepo* (Figure 2d).

**Figure 3:**
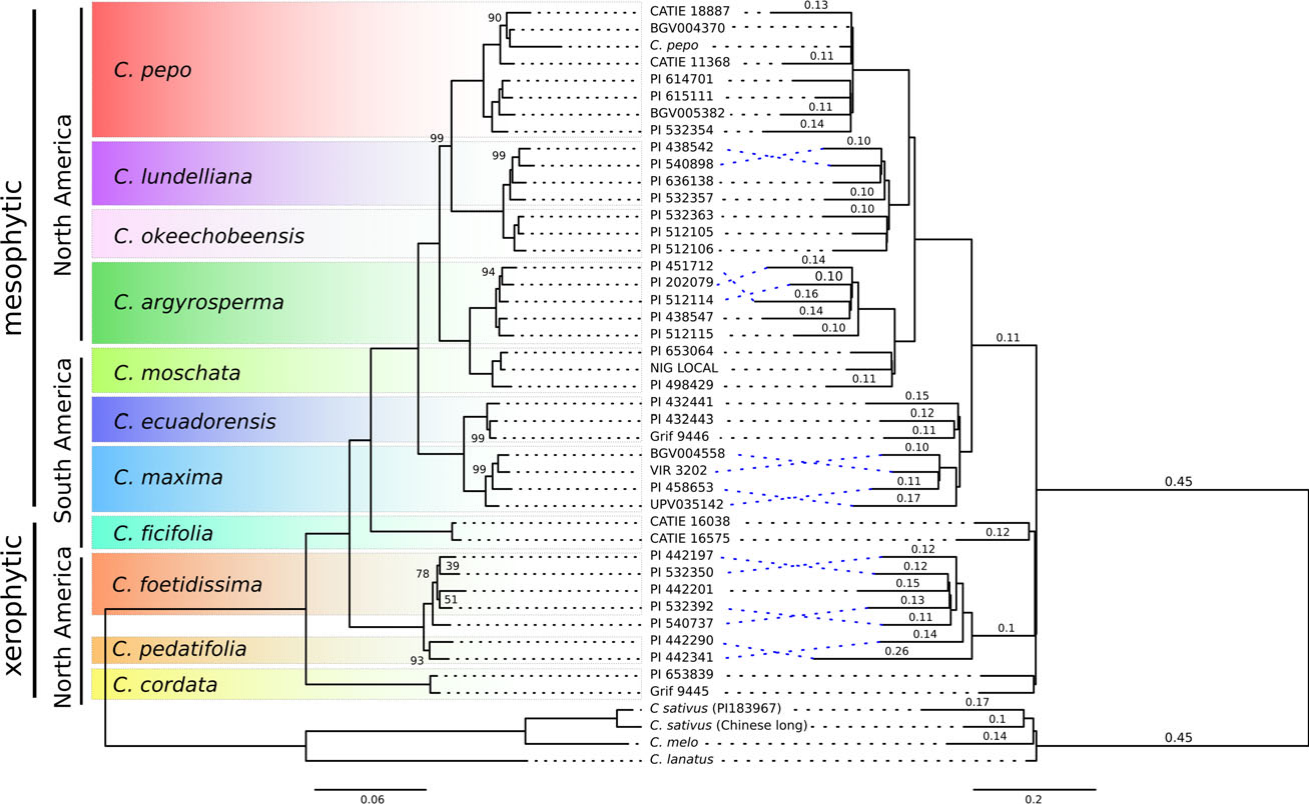
Phylogeny of the *Cucurbita* genus based on a concatenated method (left tree) or a joint estimation of gene and species trees (right tree). Left tree: branch lengths represent genetic distance and only bootstrap values lower than 100 are shown. Right tree: branch length represents proportion of duplicated genes per branch, values shown those proportions of duplicated genes higher than 0.1

A total of 40 transcriptomes were assembled by Trinity from Illumina reads for 12 species, resulting in 18 446–67 366 genes and 18 902–92 522 transcripts (Table S1). The species and gene family trees were reconstructed using Phyldog, including both the genomes and these 40 transcriptomes. Phyldog identified duplication events in the gene family trees and calculated the number of duplications per branch in the species tree. According to Phyldog, most gene families underwent a duplication event (90%) in the branch that originated the *Cucurbita* genus (Figure 3). Additionally, a maximum‐likelihood phylogeny was reconstructed using a concatenated alignment in IQ‐TREE. The topologies recovered by both methods are highly congruent. The species trees based on genomic data showed that xerophytic perennial species (*C. cordata*,* C. pedatifolia* and *C. foetidissima*) were in a basal position, whereas mesophytic annuals and short‐lived perennial species of the genus were derived from them and formed a monophyletic taxon. The only remarkable difference between both methods was the position of *C. ficifolia*: IQ‐TREE grouped it with the mesophytic species, whereas Phyldog grouped it with the xerophytic species. There are some other minor differences related with the position of some accessions within a particular species between both trees. Some of these differences are related to suspected hybrid accessions like PI540737 (between *C. pedatifolia* and *C. foetidissima*) or PI532392 (between *C. scabridifolia* and *C. foetidissima*). In general, all nodes are supported by bootstrap values close to 100 except those related with hybrids.

A GO enrichment analysis was carried out on three sets of genes: (i) single‐copy *C. pepo* genes, (ii) all duplicated *C. pepo* genes and (iii) duplicated *C. pepo* genes found to be single copy in the rest of cucurbits (i.e. melon, watermelon and cucumber). Single‐copy genes were enriched in nucleic acid metabolic processes, DNA repair, DNA replication, DNA recombination, rRNA and tRNA processing, lipid metabolism and embryo development (Table S10 and Figure S8). In the gene set found to be duplicated in all species, the most significantly enriched GO terms were transcription and translation regulation, protein metabolism, transmembrane transport, ribosome biogenesis and signal transduction. The terms enriched in the *C. pepo* exclusive duplication were NAD biosynthesis, regulation of signal transduction, mitochondrial respiratory chain, regulation of cell cycle and cell structure, intracellular protein transport, pollen and vegetative development, photosynthesis light harvesting and photoperiodism of flowering. Interestingly, other genes related to flower development were also found among the exclusively duplicated genes in *C. pepo* such as EARLY FLOWERING 4, zinc finger CONSTANS‐LIKE 3, flowering locus T, RTF1, CDF73, KNUCKLES, CTR9, flowering locus K, GID1b, FLC EXPRESSOR, FRIGIDA and FPA. Additionally, seven of 34 genes annotated as ‘similar to CONSTANS‐LIKE protein’ were exclusively duplicated in *C. pepo*, as well as five of nine genes annotated as ‘similar to FRIGIDA’.

Genome assembly and transcriptomic and genomic raw sequences are deposited in NCBI under BioProject PRJNA386743. Genome v. 4.1, genome annotation and transcriptome v. 3.0 are also available at http://bioinf.comav.upv.es.

## Discussion

In this study, we present the first description of the *C. pepo* genome. This new assembly is organized into 20 pseudomolecules, has a scaffold N50 of 1.8 Mb and is integrated with a high‐density genetic map. According to the coverage (92.1%) of the BUSCO conserved gene core set and the percentage of the RNAseq reads (91.1%) and genomic reads (99.4%) mapped against it, the current assembly covers most of the zucchini genome. The genome size inferred by *k*‐mer analysis was 283 Mb, so this assembly would constitute 93.0% of the genome. Thus, this assembly is an almost complete representation of the *C. pepo* genome.

Our results show that the *C. pepo* genome has undergone a WGD that occurred in the origin of the *Cucurbita* genus. Three independent lines of evidence support this: the topology of the gene family phylogenies, the karyotype organization and the distribution of 4DTv distances. Phyldog reconstructed the phylogeny of every gene family, and by comparing it with the species tree topology, we inferred where the duplication event likely occurred in each family. According to this analysis, most duplications occurred in the branch that separated the *Cucurbita* genus from the rest of species in the Cucurbitaceae family. The genome structure, shown by the physical location of the pairs of Zucchini paralog genes, was characterized by large syntenic regions within this species. These syntenic regions cover most of the genome and are likely to have been generated by a pseudodiplodization of an ancestral tetraploid followed by various chromosomal rearrangements. Interestingly, all species of the *Cucurbita* genus (tribe *Cucurbiteae*) present *n* = 20 chromosomes (Kocyan *et al*., 2007; Šiško *et al*., 2003/9), whereas species of *Benincaseae* tribe, which include the *Cucumis* and *Citrullus* genera, have a different chromosomal organization with *n* = 12 (melon), *n* = 11 (watermelon) or *n* = 7 (cucumber) (Kocyan *et al*., 2007). A possible polyploidy in the origin of *Cucurbita* was previously proposed based on the chromosome number and the number of isoenzyme copies (Kirkpatrick *et al*., 1985; Weeden, 1984). Despite the WGD, the size of the zucchini genome is similar to that of the other sequenced cucurbits, and the number of genes is not much higher. This suggests that most genes were deleted after the WGD event. It might well be the case that there has been a selective pressure to keep the genome size of these species within a certain range and that the maintained genes were specifically selected.

The 4DTv distribution found in *C. melo, C. sativus* and *C. lanatus* showed no evidence of a recent WGD (Garcia‐Mas *et al*., 2012; Huang *et al*., 2009). These three species present a peak in the 4DTv distribution around (0.6) that corresponds to the ancestral paleohexaploidy (γ) event that occurred in the divergence of monocotyledons and dicotyledons (~300 Mya) (Bowers *et al*., 2003). However, the 4DTv distances between paralog genes within zucchini showed shorter distances, characterized by a mode of 0.12. Thus, most paralogs seem to have been created by a recent duplication. Additionally, the 4DTv peaks found in the distribution calculated for the orthologous genes between *C. pepo* and melon, cucumber and watermelon can be used to date the Zucchini duplication. These peaks are all very close to the zucchini duplication peak. Therefore, both the 4DTv and gene family phylogenies are consistent with a duplication that occurred in the ancestral species that gave rise to the *Cucurbita* genus shortly after its split from the ancestor of *C. melo*,* C. sativus* and *C. lanatus* approximately 30 ± 4 Mya (Schaefer *et al*., 2009). The evolutionary rate derived from this time estimation is consistent with that found in other plants with a recent WGD like *Nelumbo nucifera* (4DTv = 0.17, 18 Mya) (Wang *et al*., 2013), *Glycine max* (0.057, 13 Mya) (Schmutz *et al*., 2010), *Zizania latifolia* (0.07, 13 Mya) (Guo *et al*., 2015) and *Setaria italica* (0.38, 70 Mya) (Zhang *et al*., 2012). *Populus trichocarpa* would be an exception with a much lower evolution rate (0.1, 60–65 Mya) (Tuskan *et al*., 2006), but this discrepancy could be due to the longer generation time of this plant (Smith and Donoghue, 2008). This WGD might have provided the *Cucurbita* species with a way to generate new gene functions to adapt to new habitats. In fact, this genus includes both xerophytic species, perennials adapted to dry climates and species adapted to moister or mesophytic environments, either annuals or short‐lived perennials, and extends from tropical to temperate regions of America. For instance, the duplication of the photosynthesis and flower development regulation genes found in the GO enrichment analysis could have provided mechanisms to adapt flowering to variations in temperature and the duration of days found from the southern USA to southern South America. Genes in these pathways have been implicated in the adaptation of several crops to different photoperiods and geographical adaptations (Blümel *et al*., 2015). FLOWERING LOCUS T has been described as a possible long‐distance florigenic signal in the cucurbits (Lin *et al*., 2007). The genus also includes an amazing degree of variation in morphological traits related to vine, fruit and seeds.

Consistent with previous *Cucurbita* phylogenetic studies (Jobst *et al*., 1998; Kates *et al*., 2017; Kistler *et al*., 2015; Sanjur *et al*., 2002; Wilson *et al*., 1992; Zheng *et al*., 2013), the xerophytic perennial species (*C. cordata*,* C. pedatifolia* and *C. foetidissima*) were basal to the *Cucurbita* genus. The current analysis supports the relationship among mesophytic species found by Kates *et al*. (2017), and additionally, it clarifies the clustering of the sister species *C. foetidissima* and *C. pedatifolia*, and *C. lundelliana* and *C. okeechobeensis* that were not previously resolved (Kates *et al*., 2017). The position of *C. ficifolia* remains controversial. The concatenated method clusters it as a basal species to the annual mesophytic taxa, showing a paraphyletic relationship with respect to the perennial taxa, in agreement with Wilson *et al*. (1992) and Kates *et al*. (2017). However, based on Phyldog analysis, *C. ficifolia* appears as a sister species of *C. pedatifolia* and *C. foetidissima*, in agreement with Zheng *et al*. (2013). *Cucurbita ficifolia* is a mesophytic species, but shares some morphological features with the xerophytic species. More data are needed to establish the relationship of *C. ficifolia* to the mesophytic/xerophytic species of the genus. This incongruence between trees may also be due to hybridization, as some partially fertile hybrids have been obtained between *C. ficifolia* and *C. lundelliana*,* C. foetidissima* and *C. pedatifolia* (Lira‐Saade, 1995), or it might be the result of very close speciation events.

This genome assembly constitutes a key resource for the study and breeding of the economically important *C. pepo*. Previous unpublished drafts, made available by us, of this genome have already been used in several publications related to the detection of resistance genes, the study of fruit development and the generation of molecular marker sets (Holdsworth *et al*., 2016; Martínez *et al*., 2013; Xanthopoulou *et al*., 2016). Additionally, we have assembled 40 transcriptomes for 11 species of the *Cucurbita* genus, which can be a valuable source of molecular markers as well as the foundation of comparative genomic studies.

## Experimental procedures

### Plant material, genetic material isolation and NGS sequencing

Genomic DNA was isolated from nuclei of the *Cucurbita pepo* subsp. *pepo* cultivar‐group zucchini, accession BGV004370 (also referred to as MU‐CU‐16 and held at the COMAV‐UPV GenBank, https://www.comav.upv.es). Leaves were frozen in liquid nitrogen, crushed in a mortar and put in a solution of 0.4 mm sucrose, 10 mm Tris–HCL pH 8.0, 10 mm MgCl_2_ and 5 mm β‐mercaptoethanol (20 mL per g of leaf tissue). This mixture was incubated on ice for 5 min. To eliminate debris and cellular fragments, samples were successively filtered through two filters (140 and 70 μm) and then centrifuged at 3000 ***g*** for 20 min at 4 °C. The pellet was resuspended in a solution of 0.25 mm sucrose, 10 mm Tris–HCl pH 8.0, 10 mm MgCl_2_, 1% Triton X‐100 and 5 mm β‐mercaptoethanol (1 mL per g of leaf tissue) and centrifuged again at 12 000 ***g*** for 10 min at 4 °C. Finally, the pellet was resuspended in 0.5 mL of 1.7 mm sucrose, 10 mm Tris–HCl ph 8.0, 2 mm MgCl_2_, 0.15% Triton X‐100 and 5 mm β‐mercaptoethanol and then centrifuged at 18 000 ***g*** during 1 h at 4 °C. The precipitated nuclei were resuspended in CTAB buffer, and the DNA was extracted using the CTAB protocol (Doyle and Doyle, 1990). Five genomic libraries were prepared: a 500‐bp pair‐end library and four mate‐pair libraries of 3‐, 7‐, 10‐ and 20‐Kb insert size. The first three libraries were prepared and sequenced by Macrogen (Seoul, Republic of Korea) using two Illumina Hiseq2000 lanes, one for the pair‐end library and another for the 3‐ and 7‐Kb mate‐pair libraries. The 10‐ and 20‐Kb libraries were prepared by the Boyce Thompson Institute (Ithaca, New York) using the Nextera protocol and sequenced in a single Illumina Hiseq 2000 lane.

Two different sets of transcriptomes were obtained: (i) a multitissue transcriptome from two cultivars, representing the two main *C. pepo* subspecies to assist the genome annotation and (ii) a group of 40 transcriptomes from 12 different wild and cultivated species of the *Cucurbita* genus for the phylogenetic and comparative analyses (see Table S1). In all cases, RNA was isolated using TRI reagent (Sigma), treated with DNAse and purified with a chloroform and ethanol precipitation. For the *C. pepo* transcriptome*,* two cultivars with contrasting phenotypes were used (BGV004370 or MU‐CU‐16, subsp. *pepo* cultivar‐group zucchini; and BGV005382 or UPV‐196, subsp. *ovifera* cultivar‐group scallop). RNA was extracted from different tissues: roots, leaves, apical shoots from plants in the male and female phase of development. Flower buds were collected at two early stages of flower development: mature flowers, preharvest fruits at various days after pollination, and postharvest fruits subjected to various postharvest treatments (ethylene, methylcyclopropene and cold). Equivalent amounts of RNA from each tissue were mixed into two pools, one per cultivar, and two independent cDNA libraries were prepared and sequenced in an Illumina Hiseq2000 lane by Macrogen (Seoul, Republic of Korea).

In the case of the 40 transcriptomes, in addition to the two *C. pepo* cultivars used in the multitissue transcriptome (zucchini and scallop), the analysed species included five additional genotypes of *C. pepo* (one subsp. *ovifera* (Acorn), two subsp. *pepo* (pumpkin) and two subsp. *fraterna*). Additionally, four additional domesticated taxa within the species were represented: *C. moschata* (three transcriptomes), *C. maxima* (three) and its wild ancestor *C. maxima* subsp. *andreana* Naudin (South America and Africa; one), *C. argyrosperma* Huber (southern United States and Central America; five) and *C. ficifolia* Bouché (Guatemala; two), as well as six wild species occurring in Mexico and Central and South America: the mesophytic annuals *C. ecuadorensis* Cutler & Whitaker (three), *C. okeechobeensis* (small) L.H Bailey subsp. *martinezii* (L.H. Bailey) T.C. Andres & G.P. Nabhan ex T.W. Walte (three)*,* and *C. lundelliana* L.H Bailey (four) and the xerophytic perennials *C. foetidissima* Kunth (four), *C. cordata* S. Watson (two) and *C. pedatifolia* L.H. Bailey (three). RNA was extracted exclusively from young leaves, and the cDNA libraries were prepared and sequenced in a Hiseq2000 lane at the Boyce Thompson Institute (Ithaca, New York).

### De novo genome assembly

The pair‐end and mate‐pair reads were cleaned using the *ngs_crumbs* software (code available at https://github.com/JoseBlanca/) to eliminate adapters, low‐quality bases (Phred quality <25 in a 5‐bp window), reads shorter than 50 bp and duplicated sequences. The Nextera mate‐pair reads (10‐Kb and 20‐Kb libraries) were classified by NextClip v0.8 (Leggett *et al*., 2014) according to the presence of the junction adaptor. Only the mate‐pairs for which NextClip could detect and trim the adaptor were used for the assembly. For the pre‐Nextera mate‐pair libraries, the detection and filtering of possible chimeric pairs were performed by mapping the reads against a first assembly of the genome, and only the pairs with the expected orientation and at the expected distance were kept. The implementation of this process can be found in the *classify_chimeras* and *trim_mp_chimeras* binaries of the *ngs_crumbs* software. The mitochondrial and chloroplastic reads were detected by blasting (Altschul *et al*., 1990) them against the *C. melo* organelle genomes (JF412791.1 and NC014050.1). Mitochondrial and chloroplastic reads were also included in the assembly to avoid the elimination of NUPTs (nuclear plastid DNA) and NUMTs (nuclear mitochondrial DNA segments) that could result in an artifactual fragmentation of the assembly, but only a sufficient number of randomly selected reads to reach 150X coverage. Assemblies with *k*‐mer lengths from 31 to 61 with a step‐size of 4 were made. The final assembly was carried out by SOAPdenovo2 v2.04 (Luo *et al*., 2012) using *k*‐mer size of 41. The resulting scaffolds were broken with BreakScaffolds (https://github.com/aubombarely/GenoToolBox) and reassembled with SSPACE (Boetzer *et al*., 2011). The new scaffolds were improved using SOAPdenovo2's GapCloser (Luo *et al*., 2012). Gene completeness of the assembly was assessed using BUSCO v.2 (Simão *et al*., 2015). Mitochondrial and chloroplastic scaffolds were identified using BLAST (Altschul *et al*., 1990) against the chloroplast and mitochondrial genomes of *C. melo*. Genome size was estimated from the *k*‐mer depth distribution as ∑(*d* · *k*_*d*_)/*D*, where *d* is the *k*‐mer depth, *k*_*d*_ is the number of *k*‐mers for the given depth, and *D* is the maximum *k*‐mer depth of the distribution. The leftmost part of the distribution was discarded, as it includes mostly *k*‐mers due to sequencing errors. The *k*‐mer distribution was calculated by Jellyfish (Marçais and Kingsford, 2011) using a *k*‐mer size of 31.

To detect assembly artefacts and to group scaffolds into pseudomolecules, a genetic map was built. A group of 120 individuals of an F_8_ recombinant inbreed line (RIL) (Montero‐Pau *et al*., 2017), developed through single seed descent from a previous zucchini (BGV004370) × scallop (BGV005382) F_2_ (Esteras *et al*., 2012), were genotyped by genotyping by sequencing (GBS) (Elshire *et al*., 2011). SNP calling was performed using Freebayes (Garrison and Marth, 2012), and a genetic map was constructed using the R packages R/qtl (Broman *et al*., 2003) and ASMap (Taylor and Butler, 2014) (see details in Montero‐Pau *et al*. (2017)). Scaffolds that were present in more than one linkage group in the genetic map were visually explored with Hawkeye (Schatz *et al*., 2007) and manually split. Scaffolds were ordered and oriented into pseudomolecules according to the genetic map.

### De novo transcriptome assembly

Raw reads were processed using *ngs_crumbs* software to eliminate adapter sequences, low‐quality bases (Phred quality < 25 in a 5‐bp window) and sequences shorter than 40 bp. The transcriptome was assembled with the Trinity assembler v2.0.6 (Grabherr *et al*., 2011) with default parameters. In the case of the *C. pepo* transcriptome, reads of both cultivars were merged to obtain a more comprehensive representation of the transcriptome. Additionally, reads from a previous 454‐based transcriptome (Blanca *et al*., 2011) were also included. The resulting contigs were reassembled with CAP3 (Huang and Madan, 1999) to eliminate redundancies. Low‐complexity transcripts were filtered out using *ngs_crumbs*. Trinity subcomponents were clustered using BLAST into unigene clusters using transitive clustering. Any two transcripts that shared an overlap longer than 100 bp and a similarity higher than 97% were considered to belong to the same unigene cluster. Finally, transcripts expressed less than 1% of the most highly expressed transcript in each Trinity subcomponent were filtered out using RSEM (http://deweylab.biostat.wisc.edu/rsem/). Gene completeness of the assembly was assessed using BUSCO v.2 (Simão *et al*., 2015).

### Genome annotation

Genome structural annotation was performed using Maker‐P (Campbell *et al*., 2014) (version 2.31.6) with the default parameters. The *C. pepo* transcriptome was used to train Augustus (Hoff and Stanke, 2013) (version 3.0.2) with the default parameters. SNAP (Korf, 2004) (version 2006‐07‐28) was also trained with the same data set following the instructions from the Maker‐P manual. Repetitive sequences were extracted from the genome reference using RepeatModeler (Tempel, 2012) (version 1.0.8). The *C. pepo* transcriptome, repetitive sequences and training *ab initio* gene predictor files were used for the annotation with Maker‐P. Functional annotation was performed by sequence homology search using BlastP (minimum *E*‐value of 10^−10^) with GenBank, TAIR10 and SwissProt protein data sets (downloaded 2014‐12‐21). Additionally, InterProScan (Mulder and Apweiler, 2007) was used to annotate protein domains, extending the annotation to Gene Ontology terms associated with these protein domains. Blast2GO (Conesa and Götz, 2008) was used to annotate based on a Blast search against NCBI's nr database. Functional descriptions were processed using AHRD (https://github.com/groupschoof/AHRD) with weights of 100, 50 and 30 for SwissProt, TAIR and GenBank annotation, respectively.

A structural and homology‐based approach, as described in Campbell *et al*. (2014), was used to annotate the repetitive DNA. Briefly, miniature inverted‐repeat transposable elements (MITE) and long terminal repeat (LTR) retrotransposons were collected using MITE‐Hunter (Han and Wessler, 2010), LTR‐harvest and LTR‐digest (Ellinghaus *et al*., 2008; Steinbiss *et al*., 2009). A MITE and LTR library was built after excluding false positives, and selecting representative sequences (Schnable *et al*., 2009). This library was used to mask genome sequences with RepeatMasker (Tempel, 2012), and the resulting sequences were then processed by RepeatModeler to identify other repetitive sequences.

Reference sequences of Copia and Gypsy LTR superfamilies of the retrotranscriptase gene were obtained from GyDB (Llorens *et al*., 2011). Sequences were manually aligned, and the best‐fitting nucleotide substitution model (based on Bayesian information criterion) and maximum‐likelihood tree for each superfamily were obtained using IQ‐TREE (Chernomor *et al*., 2016; Nguyen *et al*., 2015). Branch support was computed using the bootstrap ultrafast method.

### Transcriptome annotation

Transcripts were blasted against SwissProt, UniRef90 and the *Arabidopsis* proteins. Orthologues with cucumber and *Arabidopsis* were detected using a bidirectional BLAST search. The unigenes were associated with GO terms using Blast2GO software (Conesa and Götz, 2008). ORFs were predicted in the unigenes with the aid of the ESTScan software (Iseli *et al*., 1999).

### Comparative genomics

Four complete genomes of three related species belonging to the Cucurbitaceae family were included in the study for comparative genomic analyses: *Citrullus lanatus* (genome v. 1) (Guo *et al*., 2012)*, Cucumis sativus* var. *sativus* (Chinese long; v. 2) (Huang *et al*., 2009), *C. sativus* var. *hardiwickii* (Royle) Gabaer (PI 183967; v. 1) and *Cucumis melo* (v. 3.5) (Garcia‐Mas *et al*., 2012). The first three are accessible at http://www.icugi.org and the latter at http://melonomics.net. To be able to compare among genomes, the repetitive DNA characterization described above was performed in these four genomes.

Detection of gene duplications in the gene families was carried out using OrthoMCL (Fischer *et al*., 2011; Li *et al*., 2003) and OrthoMCL DB version 5 on the predicted proteomes of the five cucurbit genomes. In those cases in which more than one transcriptional variant was found for the same gene, only the longest variant was used. Differences in the functional role of the duplicated genes were assessed through GO enrichment tests using R package topGO (Alexa and Rahnenfuhrer, 2010), and REVIGO (Supek *et al*., 2011) was used to visualize the results. Transversion rates on fourfold degenerate synonymous sites (4DTv) were calculated between pairs of orthologs and paralogs using an in‐house Python script. Values were corrected for multiple substitutions (Tang *et al*., 2008).

The phylogeny (40 transcriptomes and five genomes) was reconstructed using a concatenated method and through a joint estimation of both species and gene trees carried out by Phyldog (Boussau *et al*., 2013). For the first approach, single‐copy genes that were detected using OrthoMCL and present in all cucurbit genomes were selected, and then, the corresponding *C. pepo* transcript was blasted against the 40 *Cucurbita* spp. transcriptomes. Only the blast hits with an *E*‐value higher than 10^−60^ and a match longer than 200 bp were retained. For each gene family, sequence alignments were built using an iterative refinement method implemented in MAFFT (Katoh *et al*., 2002). Alignments with less than 30 species were excluded. All resulting gene families were concatenated, and the maximum‐likelihood tree was inferred using IQ‐TREE (Nguyen *et al*., 2015) using a nucleotide substitution model for each gene (Chernomor *et al*., 2016). For each partition, the best model was selected based on the Bayesian information criterion (BIC). Branch support was obtained by bootstrap using an ultrafast method (Minh *et al*., 2013).

For the Phyldog approach, sequences were clustered in ortholog groups by blasting all *C. pepo* genes against all 40 *Cucurbita* spp. transcriptomes and the four cucurbit genomes. Blast hits with an identity lower than 70% and alignment length shorter than 200 residues were ignored. For each group, three multiple sequence alignments were obtained using Kalign (Lassmann *et al*., 2009), MUSCLE (Edgar, 2004) and MAFFT (Katoh *et al*., 2002). Alignment results were combined and evaluated with T‐Coffee (Chang *et al*., 2014; Wallace *et al*., 2006), and only alignments with an alignment score higher than 900 were kept. For each alignment, a starting tree for Phyldog was inferred using PhyML (Guindon *et al*., 2009) assuming the best nucleotide substitution model obtained by jModeltest (Posada, 2008). Phyldog (Boussau *et al*., 2013) was then used to simultaneously infer species and gene trees and to detect gene duplication events.

## Supporting information


**Figure S1** Distribution of sequences of *k*‐mer size 41 for different levels of coverage.

**Figure S2** Distribution of N50 for contigs (A) and scaffolds (B) for different *k*‐mer size values.

**Figure S3** Correlation between genetic and physical distances for each pseudochromosome. Color scale represents fraction of repetitive DNA.

**Figure S4** Summary of the structural annotation of *C. pepo* genome.

**Figure S5** Transcriptome GO annotation statistics A) by levels and B) at level 6.

**Figure S6** Genome GO annotation statistics (A) by levels and (B) at level 6.

**Figure S7** Repetitive elements. Fraction of the genome covered by different types of repetitive elements in *C. pepo and* four *Cucurbita* genomes (A). Maximum likelihood phylogenetic trees of *C. pepo* elements of *Copia* (B) and *Gypsy* (C) LTR superfamilies based on a fragment of the reverse transcriptase.

**Figure S8** Results of the GO enrichment test. Treemaps for the results of the GO enrichment tests on single‐copy genes in *Cucurbita pepo,* all duplicated genes in *C. pepo* and genes that are duplicated in *C. pepo* but not in other cucurbit genomes. The area of the rectangles represents the negative logarithm of the enrichment test FDR.

**Table S1** Accessions of domesticated and wild *Cucurbita* spp. used for transcriptomic and phylogenetic analyses. The number of reads used for assembly the transcriptomes, and number of genes and transcripts obtained are also shown.

**Table S2** NGS library statistics. Numbers of raw reads, percentage of nucleotides over 30 quality, coverage, % of reads filtered out during the cleaning process, % of reads without adaptor, % of chimeric reads, number of cleaned reads, coverage of cleaned reads, and percentage of nucleotides over 30 quality in the clean reads.

**Table S3** Scaffolds of genome assembly v.3.2. containing chloroplastic and mitochondrial regions. The pseudochromosomes were built from the version 3.2 scaffolds.

**Table S4** Genome v.4.1 pseudochromosome configuration. The order, orientation and size of genome v. 3.2 scaffolds grouped in each pseudochromosome is shown. Equivalence of pseudochromosomes and linkage groups of Montero‐Pau *et al*. (2017) genetic map is also shown.

**Table S5** Summary of repetitive elements found in *Cucurbita pepo, Cucumis melo, Cucumis sativus* and *Citrullus lanatus*. All results are expressed in bp.

**Table S6** Gene family (orthogroups and paralogs in OrthoMCL) identification.

**Table S7** List of genes that are single copy in *Cucurbita pepo*. Predicted functions are also shown.

**Table S8** List of genes that are duplicated in *Cucurbita pepo*. Predicted functions are also shown.

**Table S9** List of genes that are duplicated in *Cucurbita pepo* but not in *Cucumis melo, Cucumis sativus* or *Citrullus lanatus*. Predicted functions are also shown.

**Table S10** GO term enrichment tests. The results are shown for single‐copy genes in *Cucurbita pepo,* duplicated genes, and genes that are exclusively duplicated in *C. pepo* when compared with other cucurbit genomes.
