# Supplementary figures and images for: De novo assembly of the zucchini genome reveals a whole‐genome duplication associated with the origin of the Cucurbita genus
Source: Plant Biotechnol J. 2017 Dec 4;16(6):1161–71. doi: 10.1111/pbi.12860 (PMC5978595; doi:10.1111/pbi.12860)

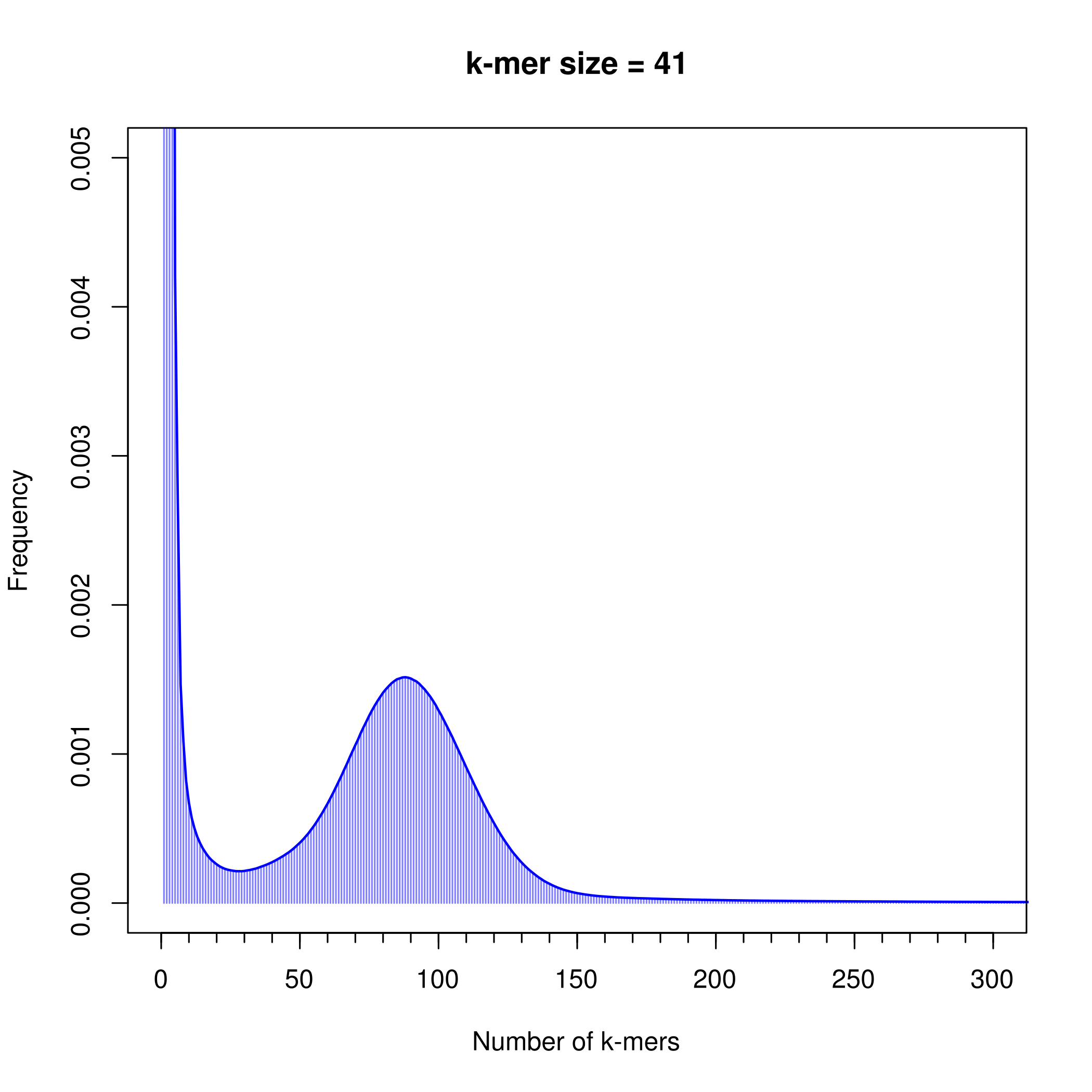

Supplement: Supplementary file 1 — Figure S1 Distribution of sequences of k‐mer size 41 for different levels of coverage. [file PBI-16-1161-s017.png]

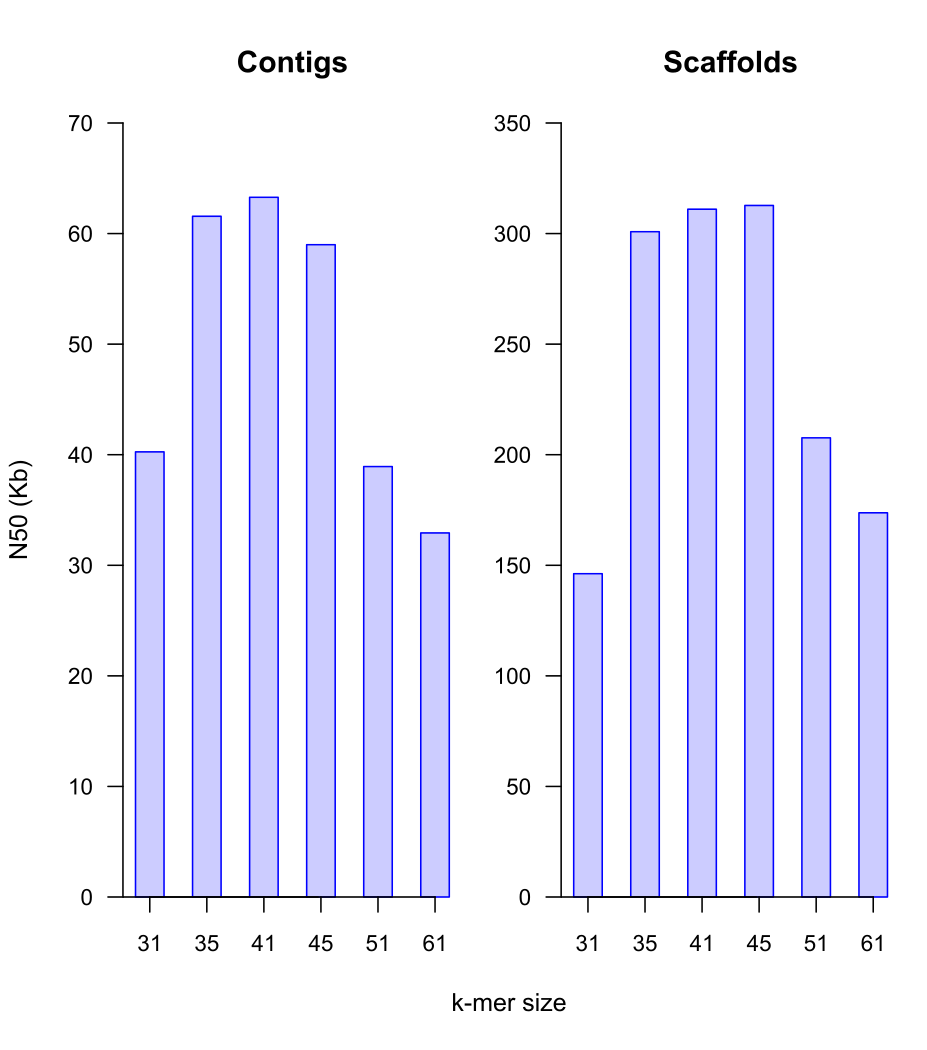

Supplement: Supplementary file 2 — Figure S2 Distribution of N50 for contigs (A) and scaffolds (B) for different k‐mer size values. [file PBI-16-1161-s018.png]

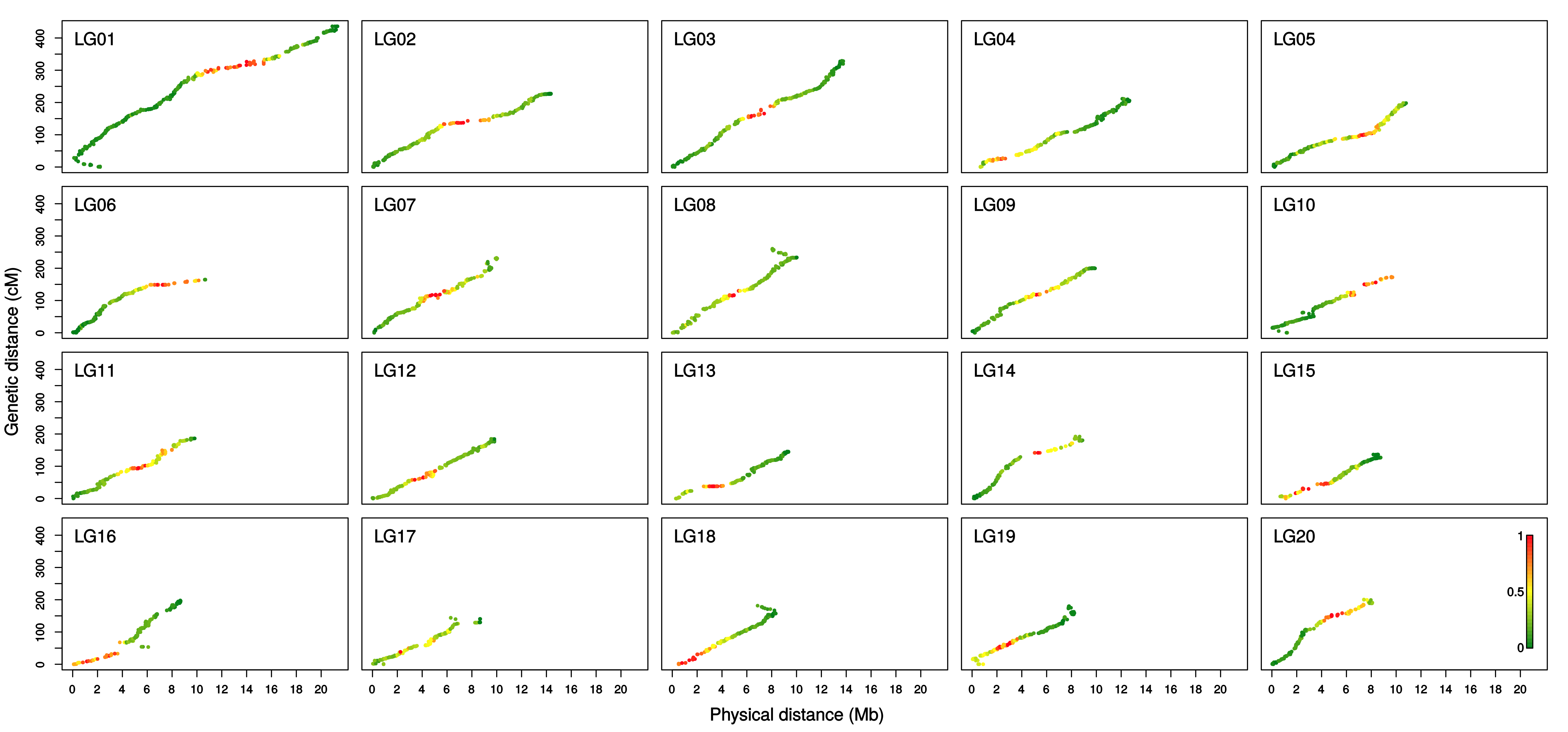

Supplement: Supplementary file 3 — Figure S3 Correlation between genetic and physical distances for each pseudochromosome. Color scale represents fraction of repetitive DNA. [file PBI-16-1161-s016.png]

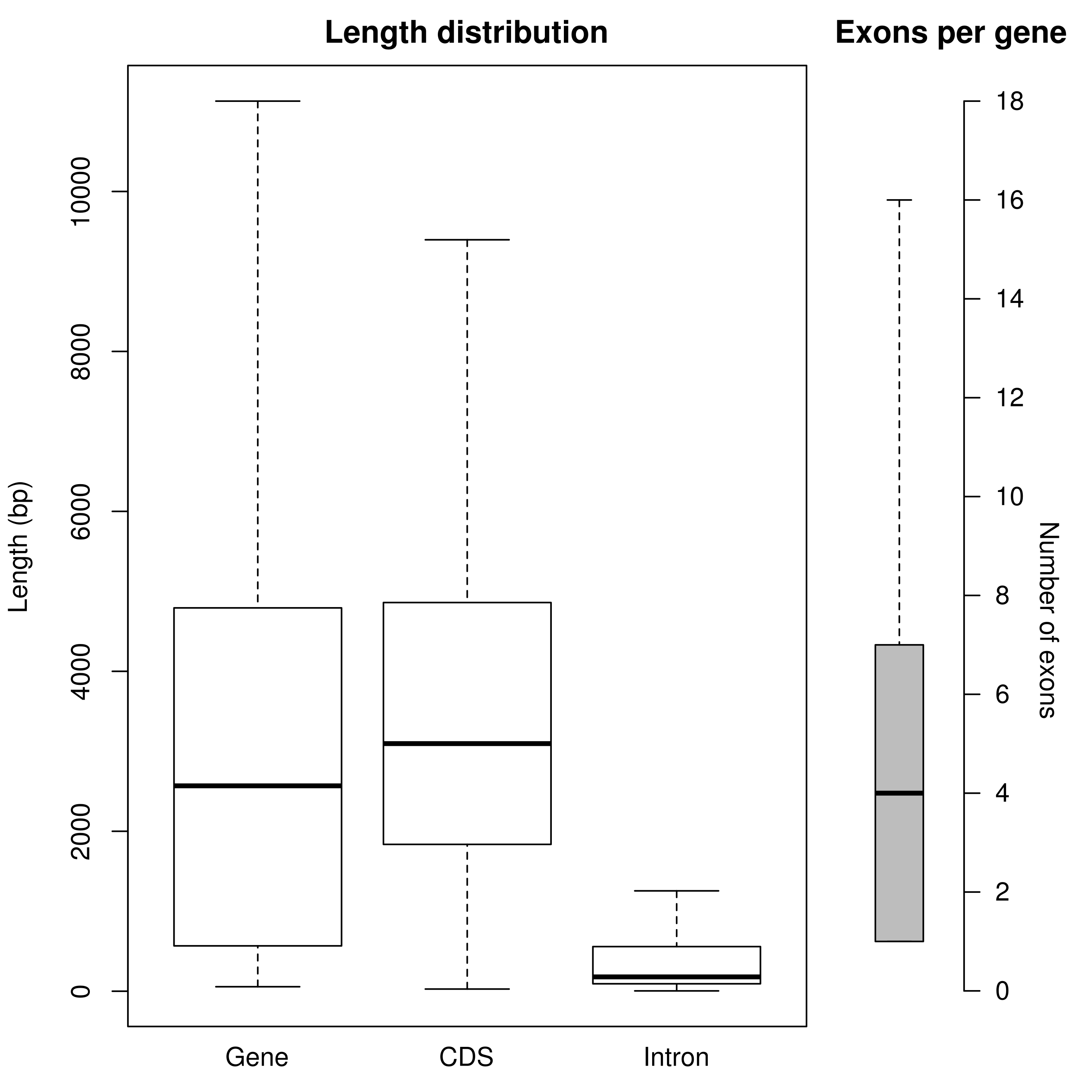

Supplement: Supplementary file 4 — Figure S4 Summary of the structural annotation of C. pepo genome. [file PBI-16-1161-s015.png]

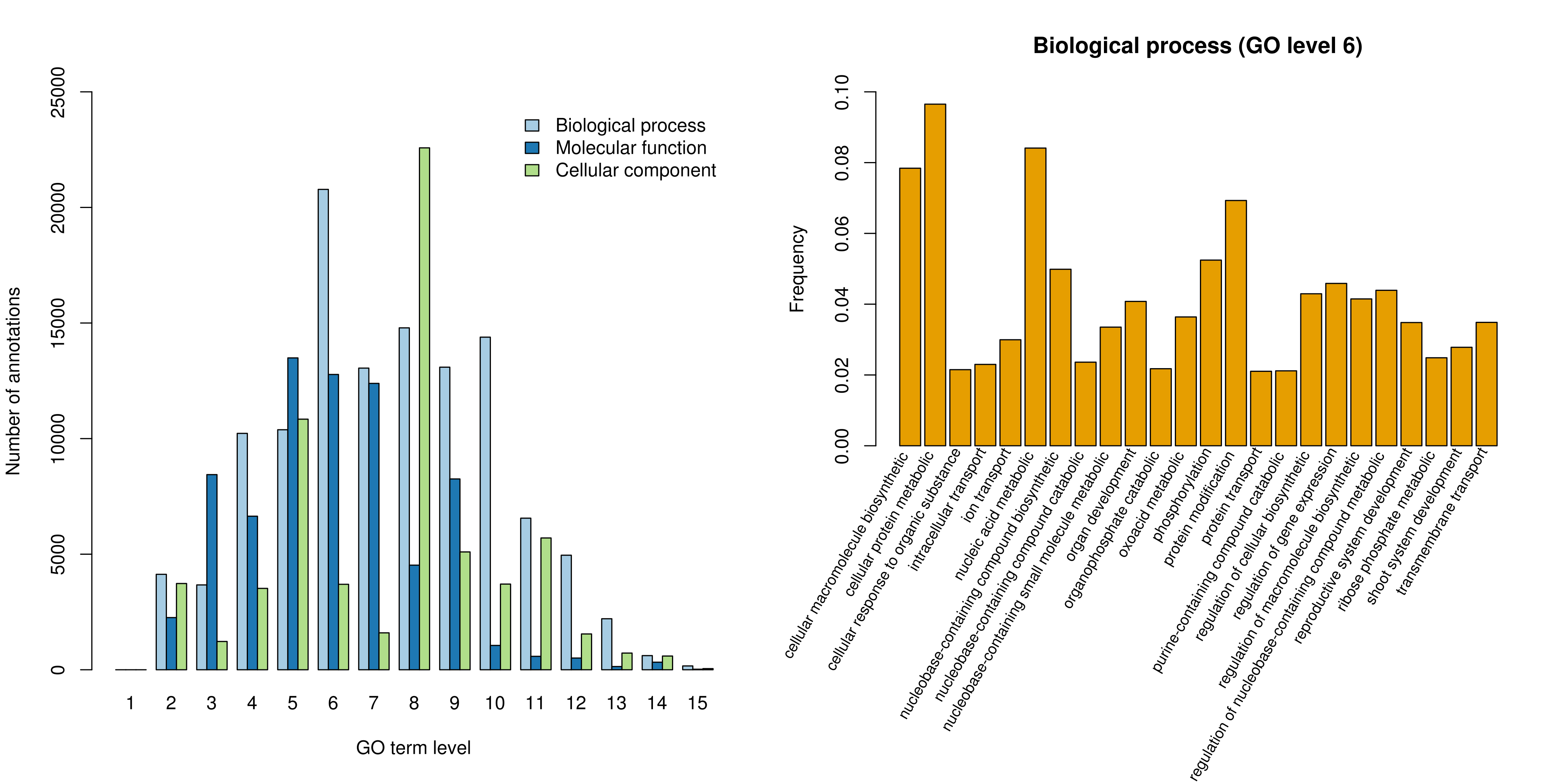

Supplement: Supplementary file 5 — Figure S5 Transcriptome GO annotation statistics A) by levels and B) at level 6. [file PBI-16-1161-s014.png]

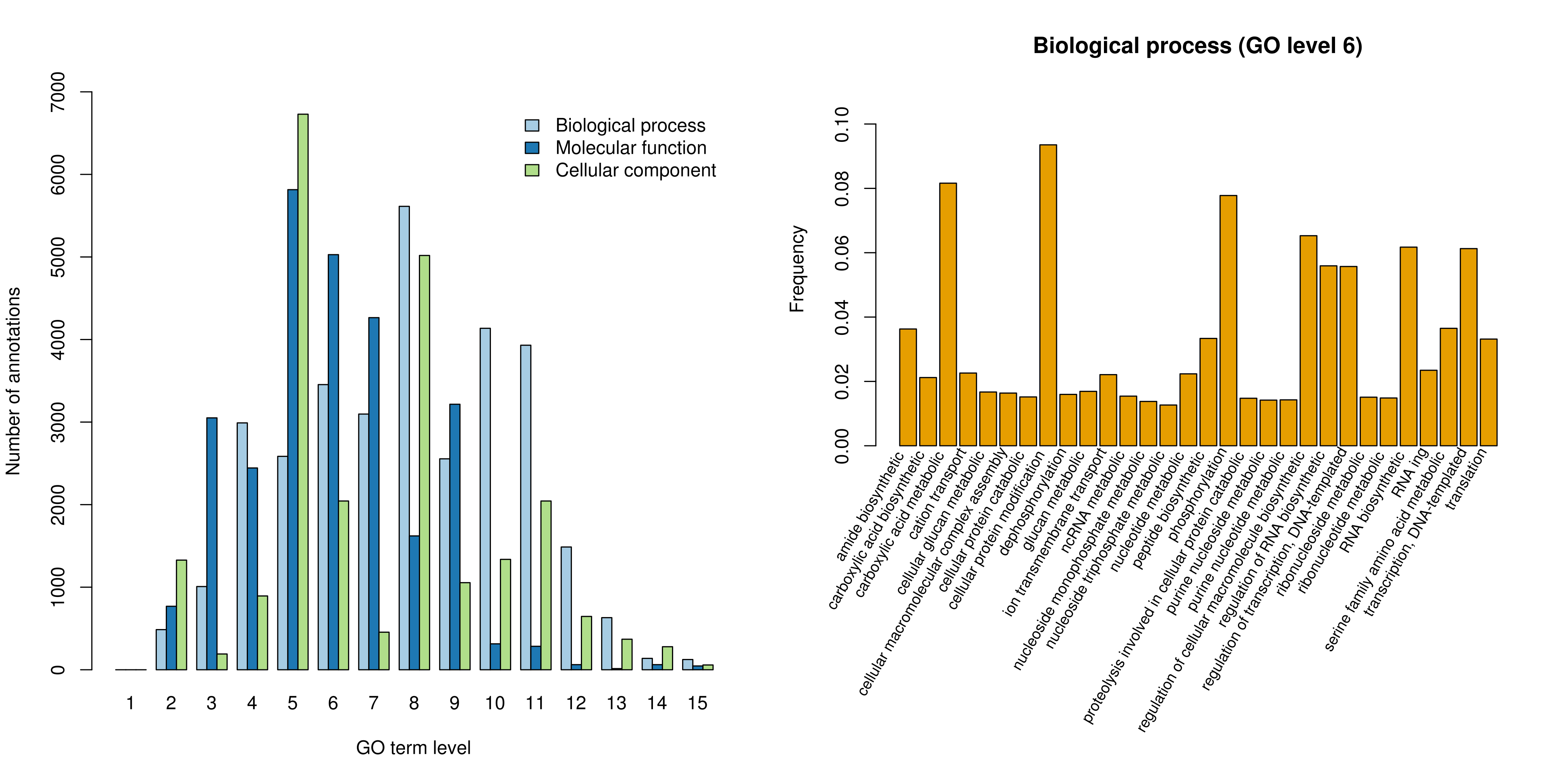

Supplement: Supplementary file 6 — Figure S6 Genome GO annotation statistics (A) by levels and (B) at level 6. [file PBI-16-1161-s001.png]

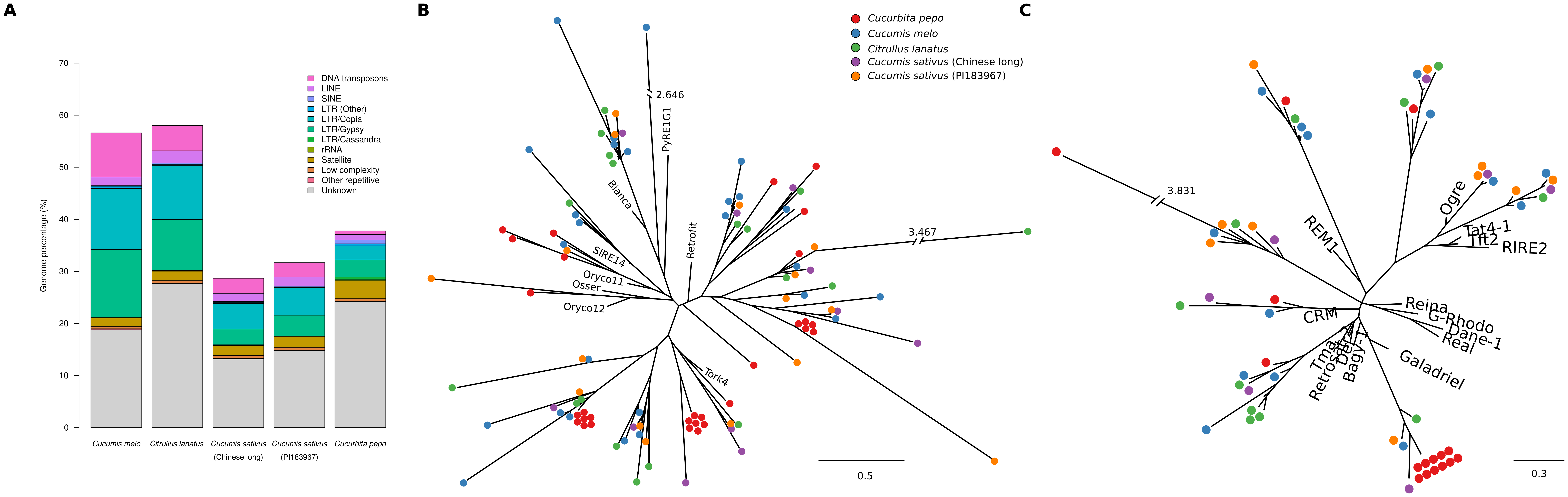

Supplement: Supplementary file 7 — Figure S7 Repetitive elements. Fraction of the genome covered by different types of repetitive elements in C. pepo and four Cucurbita genomes (A). Maximum likelihood phylogenetic trees of C. pepo elements of Copia (B) and Gypsy (C) LTR superfamilies based on a fragment of the reverse transcriptase. [file PBI-16-1161-s002.png]

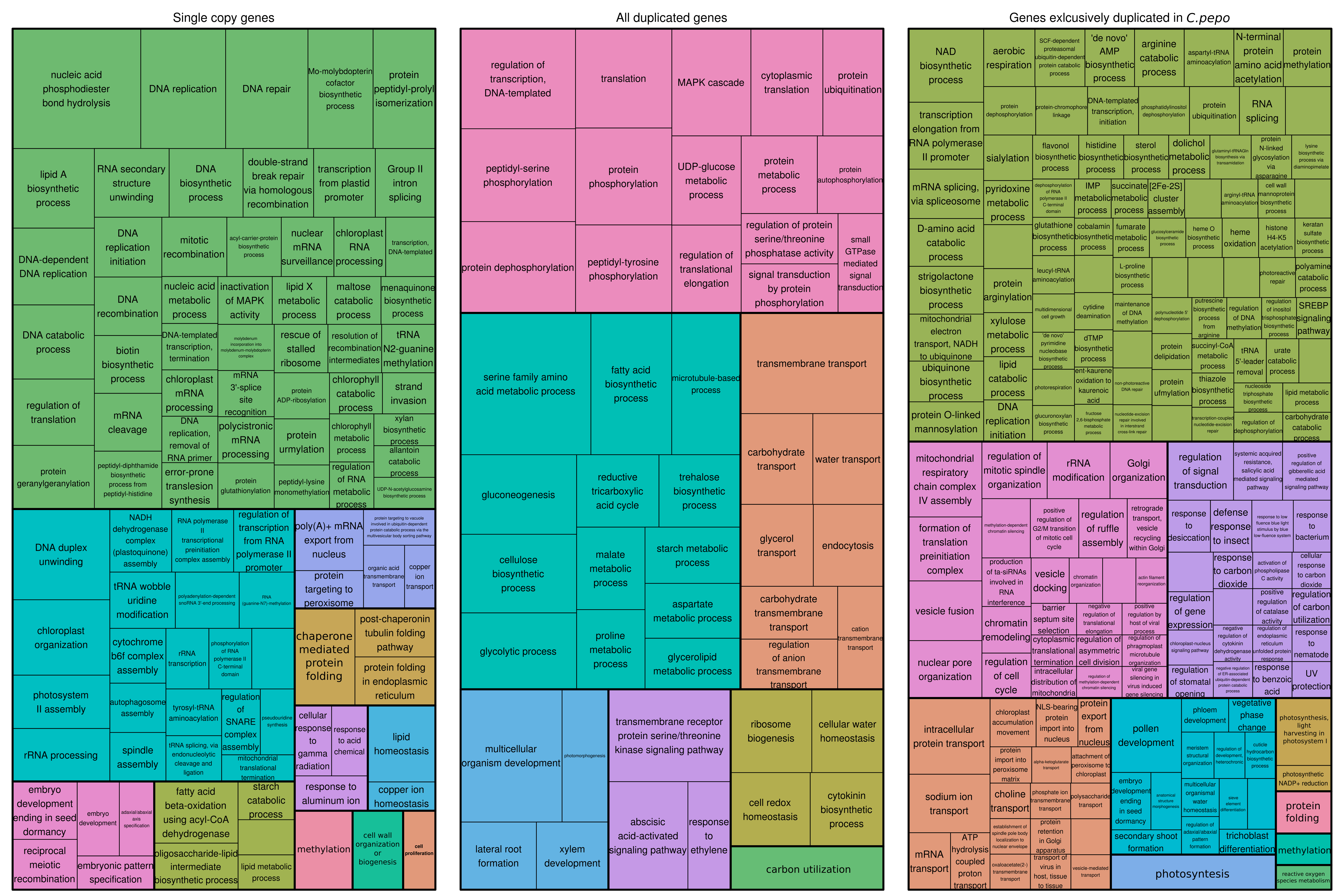

Supplement: Supplementary file 8 — Figure S8 Results of the GO enrichment test. Treemaps for the results of the GO enrichment tests on single‐copy genes in Cucurbita pepo, all duplicated genes in C. pepo and genes that are duplicated in C. pepo but not in other cucurbit genomes. The area of the rectangles represents the negative logarithm of the enrichment test FDR. [file PBI-16-1161-s003.png]
