# Supplementary material for: De novo assembly of the zucchini genome reveals a whole‐genome duplication associated with the origin of the Cucurbita genus
Source: Plant Biotechnol J. 2017 Dec 4;16(6):1161–71. doi: 10.1111/pbi.12860 (PMC5978595; doi:10.1111/pbi.12860)
Supplement: Supplementary file 9 — Table S1 Accessions of domesticated and wild Cucurbita spp. used for transcriptomic and phylogenetic analyses. The number of reads used for assembly the transcriptomes, and number of genes and transcripts obtained are also shown. [file PBI-16-1161-s004.docx]

Supplementary Table 1. Accessions of domesticated and wild *Cucurbita* spp. used for transcriptomic and phylogenetic analyses. Number of reads used for assembly the transcriptomes, and number of genes and transcripts obtained are also shown.

| **Code** | **Donor bank** | **Species** | **Subspecies (cultivar-group)** | **Country** | **Observations*** | **Number of reads** | **Number of genes** | **Number of transcripts** |
| --- | --- | --- | --- | --- | --- | --- | --- | --- |
| BGV004370 | COMAV | *C. pepo* | *pepo* (Zucchini) | Spain |  | 1,892,580 | 18,446 | 18,902 |
| BGV005382 | COMAV | *C. pepo* | *ovifera* (Scallop) | Spain |  | 3,858,970 | 30,202 | 31,603 |
| PI 615111 | USDA | *C. pepo* | *ovifera* (Acorn) | USA |  | 7,305,712 | 43,585 | 47,003 |
| CATIE 18887 | CATIE | *C. pepo* | *pepo* (Pumpkin) | Mexico |  | 9,043,764 | 48,101 | 52,134 |
| CATIE 11368 | CATIE | *C. pepo* | *pepo* (Pumpkin) | Guatemala |  | 7,881,248 | 44,956 | 48,891 |
| PI 532354 | USDA | *C. pepo* | *fraterna* | Mexico |  | 14,574,156 | 54,631 | 63,051 |
| PI 614701 | USDA | *C. pepo* | *ozarkana* | USA | Reclassified as fraterna based on fruit traits | 11,131,674 | 41,507 | 45,626 |
| Nigerian Local | Seed company | *C. moschata* |  | Nigeria |  | 11,013,414 | 46,834 | 51,731 |
| PI 498429 | USDA | *C. moschata* |  | Colombia |  | 6,371,004 | 47,871 | 52,874 |
| PI 653064 | USDA | *C. moschata* |  | Nigeria |  | 8,793,992 | 40,308 | 43,531 |
| BGV004558 | COMAV | *C. maxima* |  | Argentina |  | 7,687,590 | 49,103 | 55,178 |
| VIR 3202 | VIR | *C. maxima* |  | Chile |  | 6,941,722 | 45,663 | 49,882 |
| UPV035142 | COMAV | *C. maxima* |  | Angola |  | 8,444,322 | 51,958 | 59,291 |
| PI 458653 | USDA | *C. maxima* | *andreana* | Argentina |  | 9,186,758 | 39,317 | 42,617 |
| PI 512115 | USDA | *C. argyrosperma* | *argyrosperma* | Guatemala |  | 8,431,320 | 44,179 | 50,119 |
| PI 451712 | USDA | *C. argyrosperma* | *argyrosperma* | USA |  | 17,537,688 | 56,350 | 69,160 |
| PI 438547 | USDA | *C. argyrosperma* |  | Belize |  | 13,654,318 | 50,299 | 58,029 |
| PI 202079 | USDA | *C. argyrosperma* | *argyrosperma* | Mexico |  | 10,173,060 | 43,676 | 48,547 |
| PI 512114 | USDA | *C. argyrosperma* | *argyrosperma* | Nicaragua |  | 8,312,838 | 37,028 | 40,099 |
| CATIE 16038 | CATIE | *C. ficifolia* |  | Guatemala |  | 11,304,986 | 40,213 | 43,269 |
| CATIE 16575 | CATIE | *C. ficifolia* |  | Guatemala |  | 11,415,514 | 54,537 | 60,549 |
| PI 432441 | USDA | *C. ecuadorensis* |  | Ecuador |  | 7,045,030 | 39,920 | 42,730 |
| PI 432443 | USDA | *C. ecuadorensis* |  | Ecuador |  | 5,165,038 | 43,184 | 46,323 |
| Grif 9446 | USDA | *C. ecuadorensis* |  | Ecuador |  | 11,424,844 | 49,330 | 54,999 |
| PI 532363 | USDA | *C. okeechobeensis* | *martinezii* | Mexico |  | 9,208,076 | 39,684 | 42,810 |
| PI 512105 | USDA | *C. okeechobeensis* | *martinezii* | Mexico |  | 5,670,572 | 42,321 | 45,035 |
| PI 512106 | USDA | *C. okeechobeensis* | *martinezii* | Mexico |  | 4,097,656 | 42,418 | 45,173 |
| PI 438542 | USDA | *C. lundelliana* |  | Belize |  | 10,515,350 | 45,096 | 49,266 |
| PI 532357 | USDA | *C. lundelliana* |  | Mexico |  | 6,957,802 | 40,933 | 44,365 |
| PI 636138 | USDA | *C. lundelliana* |  | Belize |  | 11,478,416 | 52,299 | 57,111 |
| PI 540898 | USDA | *C. lundelliana* |  | Honduras |  | 7,510,758 | 53,599 | 59,801 |
| PI 442197 | USDA | *C. foetidissima* |  | Mexico |  | 7,893,284 | 50,770 | 58,496 |
| PI 532350 | USDA | *C. foetidissima* |  | Mexico |  | 8,023,710 | 39,222 | 44,100 |
| PI 442201 | USDA | *C. foetidissima* |  | Mexico | Possible hybrid based on morphology | 12,257,620 | 54,205 | 65,217 |
| PI 532392 | USDA | *C.* x *scabridifolia* |  | Mexico | Hybrid C. foetidissima x C. scabridifolia | 9,427,190 | 42,885 | 48,184 |
| PI 653839 | USDA | *C. cordata* |  | Mexico |  | 5,055,088 | 38,784 | 41,705 |
| Grif 9445 | Seed company | *C. cordata* |  | Mexico |  | 5,756,634 | 33,530 | 36,153 |
| PI 442341 | USDA | *C. pedatifolia* |  | Mexico |  | 8,712,776 | 39,572 | 45,615 |
| PI 442290 | USDA | *C. pedatifolia* |  | Mexico |  | 27,822,108 | 67,366 | 92,522 |
| PI 540737 | USDA | *C. pedatifolia* |  | Mexico | Hybrid C. pedatifolia x C. foetidissima | 7,485,656 | 35,562 | 39,306 |

* These accessions were morphologically characterized to confirm their taxonomic classification. Some of them were proved to be misclassified.
