# Supplementary material for: De novo assembly of the zucchini genome reveals a whole‐genome duplication associated with the origin of the Cucurbita genus
Source: Plant Biotechnol J. 2017 Dec 4;16(6):1161–71. doi: 10.1111/pbi.12860 (PMC5978595; doi:10.1111/pbi.12860)
Supplement: Supplementary file 10 — Table S2 NGS library statistics. Numbers of raw reads, percentage of nucleotides over 30 quality, coverage, % of reads filtered out during the cleaning process, % of reads without adaptor, % of chimeric reads, number of cleaned reads, coverage of cleaned reads, and percentage of nucleotides over 30 quality in the clean reads. [file PBI-16-1161-s005.docx]

Supplementary Table 2. NGS library statistics. Numbers of raw reads, percentage of nucleotides over a 30 quality, coverage, % of reads filtered out during the cleaning process, % of reads without adaptor, % of chimeric reads, number of cleaned reads, coverage of cleaned reads, and percentage of nucleotides over a 30 quality in the clean reads.

| **Library** | **# Raw reads** | **Q30** | **Coverage** | **% Cleaned** | **% Category D** | **% Chimeric** | **# Filtered reads** | **Coverage clean** | **% Q30**  **clean** |
| --- | --- | --- | --- | --- | --- | --- | --- | --- | --- |
| Pair-end | 882,803,080 | 88.60 | 254 | 43.00 | -- | -- | 503,219,464 | 145 | 99.98 |
| 3 Kb | 186,878,960 | 91.32 | 54 | 49.60 | -- | 24.53 | 71,085,422 | 31 | 99.96 |
| 7 Kb | 159,602,336 | 90.74 | 46 | 73.67 | -- | 26.94 | 30,700,824 | 13 | 99.94 |
| 10 Kb | 149,980,526 | 89.62 | 65 | 48.40 | 28.59 | 3.55 | 53,381,028 | 23 | 99.986 |
| 20 Kb | 143,080,152 | 88.14 | 62 | 69.22 | 30.12 | 4.75 | 30,047,048 | 13 | 99.82 |
