# Supplementary material for: De novo assembly of the zucchini genome reveals a whole‐genome duplication associated with the origin of the Cucurbita genus
Source: Plant Biotechnol J. 2017 Dec 4;16(6):1161–71. doi: 10.1111/pbi.12860 (PMC5978595; doi:10.1111/pbi.12860)
Supplement: Supplementary file 11 — Table S3 Scaffolds of genome assembly v.3.2. containing chloroplastic and mitochondrial regions. The pseudochromosomes were built from the version 3.2 scaffolds. [file PBI-16-1161-s006.docx]

Supplementary Table 3. Scaffolds of genome assembly v.3.2. containing chloroplastic and mitochondrial regions. The pseudochromosomes were build out of the version 3.2 scaffolds

| **Mitochondrion** | | | **Chloroplast** |
| --- | --- | --- | --- |
| CP32_scaffold000204 | CP32_scaffold000212 | CP32_scaffold000255 | CP32_scaffold000227 |
| CP32_scaffold000205 | CP32_scaffold000213 | CP32_scaffold000256 | CP32_scaffold000415 |
| CP32_scaffold000206 | CP32_scaffold000214 | CP32_scaffold000257 | CP32_scaffold000421 |
| CP32_scaffold000207 | CP32_scaffold000215 | CP32_scaffold000258 | CP32_scaffold000922 |
| CP32_scaffold000208 | CP32_scaffold000216 | CP32_scaffold000259 | CP32_scaffold001585 |
| CP32_scaffold000209 | CP32_scaffold000217 | CP32_scaffold000260 | CP32_scaffold002404 |
| CP32_scaffold000210 | CP32_scaffold000218 | CP32_scaffold000261 | CP32_scaffold002426 |
| CP32_scaffold000211 | CP32_scaffold000219 | CP32_scaffold000262 | CP32_scaffold002979 |
| CP32_scaffold000212 | CP32_scaffold000220 | CP32_scaffold000263 | CP32_scaffold003103 |
| CP32_scaffold000213 | CP32_scaffold000221 | CP32_scaffold000264 | CP32_scaffold003469 |
| CP32_scaffold000214 | CP32_scaffold000222 | CP32_scaffold000265 | CP32_scaffold003889 |
| CP32_scaffold000215 | CP32_scaffold000223 | CP32_scaffold000266 | CP32_scaffold005778 |
| CP32_scaffold000216 | CP32_scaffold000224 | CP32_scaffold000267 | CP32_scaffold007510 |
| CP32_scaffold000217 | CP32_scaffold000225 | CP32_scaffold000268 |  |
| CP32_scaffold000218 | CP32_scaffold000226 | CP32_scaffold000269 |  |
| CP32_scaffold000219 | CP32_scaffold000227 | CP32_scaffold000270 |  |
| CP32_scaffold000220 | CP32_scaffold000228 | CP32_scaffold000271 |  |
| CP32_scaffold000221 | CP32_scaffold000229 | CP32_scaffold000272 |  |
| CP32_scaffold000222 | CP32_scaffold000230 | CP32_scaffold000273 |  |
| CP32_scaffold000223 | CP32_scaffold000231 | CP32_scaffold000274 |  |
| CP32_scaffold000224 | CP32_scaffold000232 | CP32_scaffold000275 |  |
| CP32_scaffold000225 | CP32_scaffold000233 | CP32_scaffold000276 |  |
| CP32_scaffold000226 | CP32_scaffold000234 | CP32_scaffold000277 |  |
| CP32_scaffold000227 | CP32_scaffold000235 | CP32_scaffold000278 |  |
| CP32_scaffold000228 | CP32_scaffold000236 | CP32_scaffold000279 |  |
| CP32_scaffold000229 | CP32_scaffold000237 | CP32_scaffold000280 |  |
| CP32_scaffold000230 | CP32_scaffold000238 | CP32_scaffold000281 |  |
| CP32_scaffold000231 | CP32_scaffold000239 | CP32_scaffold000282 |  |
| CP32_scaffold000232 | CP32_scaffold000240 | CP32_scaffold000283 |  |
| CP32_scaffold000233 | CP32_scaffold000241 | CP32_scaffold000284 |  |
| CP32_scaffold000234 | CP32_scaffold000242 | CP32_scaffold000285 |  |
| CP32_scaffold000235 | CP32_scaffold000243 | CP32_scaffold000286 |  |
| CP32_scaffold000236 | CP32_scaffold000244 | CP32_scaffold000287 |  |
| CP32_scaffold000237 | CP32_scaffold000245 | CP32_scaffold000288 |  |
| CP32_scaffold000238 | CP32_scaffold000246 | CP32_scaffold000289 |  |
| CP32_scaffold000239 | CP32_scaffold000247 | CP32_scaffold000290 |  |
| CP32_scaffold000240 | CP32_scaffold000248 | CP32_scaffold000291 |  |
| CP32_scaffold000241 | CP32_scaffold000249 | CP32_scaffold000292 |  |
| CP32_scaffold000242 | CP32_scaffold000250 | CP32_scaffold000293 |  |
| CP32_scaffold000243 | CP32_scaffold000251 | CP32_scaffold000294 |  |
| CP32_scaffold000244 | CP32_scaffold000252 | CP32_scaffold000295 |  |
| CP32_scaffold000245 |  |  |  |
