# Supplementary material for: De novo assembly of the zucchini genome reveals a whole‐genome duplication associated with the origin of the Cucurbita genus
Source: Plant Biotechnol J. 2017 Dec 4;16(6):1161–71. doi: 10.1111/pbi.12860 (PMC5978595; doi:10.1111/pbi.12860)
Supplement: Supplementary file 12 — Table S4 Genome v.4.1 pseudochromosome configuration. The order, orientation and size of genome v. 3.2 scaffolds grouped in each pseudochromosome is shown. Equivalence of pseudochromosomes and linkage groups of Montero‐Pau et al. (2017) genetic map is also shown. [file PBI-16-1161-s007.docx]

Supplementary Table 4. Genome v.4.1 pseudochromosomes configuration. The order, orientation and size of genome v. 3.2 scaffolds grouped in each pseudochromosomes is shown. Equivalence of pseudochromosomes and linkage groups of Montero-Pau et al. (2016) genetic map is also shown.

| **Genome v.4.1 pseudochromosome** | **Genetic map linkage group in Montero-Pau et al. 2016** | **Scaffold order** | **Scaffold name** | **Scaffold size** | **Scaffold orientation** |
| --- | --- | --- | --- | --- | --- |
| CP4.1LG01 | LG01 | 1 | CP32_scaffold000010 | 3,883,160 | reverse |
|  |  | 2 | CP32_scaffold000175 | 233,039 | reverse |
|  |  | 3 | CP32_scaffold000046 | 1,602,464 | reverse |
|  |  | 4 | CP32_scaffold000051 | 1,450,516 | reverse |
|  |  | 5 | CP32_scaffold000040 | 1,852,384 | reverse |
|  |  | 6 | CP32_scaffold000144 | 331,714 | forward |
|  |  | 7 | CP32_scaffold000084 | 741,359 | forward |
|  |  | 8 | CP32_scaffold000063 | 1,087,272 | forward |
|  |  | 9 | CP32_scaffold000111 | 460,681 | forward |
|  |  | 10 | CP32_scaffold000233 | 109,860 | reverse |
|  |  | 11 | CP32_scaffold000078 | 832,487 | forward |
|  |  | 12 | CP32_scaffold000181 | 222,517 | reverse |
|  |  | 13 | CP32_scaffold000059 | 1,137,078 | reverse |
|  |  | 14 | CP32_scaffold000079 | 790,494 | forward |
|  |  | 15 | CP32_scaffold000087 | 658,871 | reverse |
|  |  | 16 | CP32_scaffold000105 | 507,762 | forward |
|  |  | 17 | CP32_scaffold000032 | 2,241,395 | forward |
|  |  | 18 | CP32_scaffold000024 | 2,525,860 | reverse |
|  |  | 19 | CP32_scaffold000091 | 633,856 | forward |
| CP4.1LG02 | LG02 | 1 | CP32_scaffold000001 | 6,123,784 | reverse |
|  |  | 2 | CP32_scaffold000128 | 403,365 | reverse |
|  |  | 3 | CP32_scaffold000099 | 554,539 | forward |
|  |  | 4 | CP32_scaffold000166 | 257,955 | forward |
|  |  | 5 | CP32_scaffold000090 | 642,088 | undefined |
|  |  | 6 | CP32_scaffold000076 | 841,349 | undefined |
|  |  | 7 | CP32_scaffold000055 | 1,317,493 | reverse |
|  |  | 8 | CP32_scaffold000162 | 279,451 | reverse |
|  |  | 9 | CP32_scaffold000208 | 154,271 | forward |
|  |  | 10 | CP32_scaffold000374 | 40,295 | undefined |
|  |  | 11 | CP32_scaffold000140 | 338,305 | forward |
|  |  | 12 | CP32_scaffold000062 | 1,102,807 | reverse |
|  |  | 13 | CP32_scaffold002675 | 2,094 | undefined |
|  |  | 14 | CP32_scaffold000169 | 245,895 | reverse |
|  |  | 15 | CP32_scaffold000041 | 1,808,812 | reverse |
|  |  | 16 | CP32_scaffold000168 | 248,911 | forward |
| CP4.1LG03 | LG03 | 1 | CP32_scaffold000038 | 1,877,481 | reverse |
|  |  | 2 | CP32_scaffold000122 | 422,066 | reverse |
|  |  | 3 | CP32_scaffold000195 | 188,662 | forward |
|  |  | 4 | CP32_scaffold000019 | 3,089,961 | reverse |
|  |  | 5 | CP32_scaffold000117 | 430,983 | reverse |
|  |  | 6 | CP32_scaffold000210 | 151,564 | forward |
|  |  | 7 | CP32_scaffold000163 | 278,935 | forward |
|  |  | 8 | CP32_scaffold000177 | 230,436 | undefined |
|  |  | 9 | CP32_scaffold000118 | 428,730 | forward |
|  |  | 10 | CP32_scaffold000187 | 210,711 | forward |
|  |  | 11 | CP32_scaffold000006 | 4,754,185 | reverse |
|  |  | 12 | CP32_scaffold000044 | 1,697,700 | reverse |
| CP4.1LG04 | LG18 + LG20 | 1 | CP32_scaffold000027 | 2,403,537 | reverse |
|  |  | 2 | CP32_scaffold000207 | 155,442 | forward |
|  |  | 3 | CP32_scaffold000002 | 5,399,389 | reverse |
|  |  | 4 | CP32_scaffold000025 | 2,522,528 | reverse |
|  |  | 5 | CP32_scaffold000033 | 2,224,244 | forward |
| CP4.1LG05 | LG04 | 1 | CP32_scaffold000066 | 995,157 | reverse |
|  |  | 2 | CP32_scaffold000180 | 223,211 | reverse |
|  |  | 3 | CP32_scaffold000143 | 334,696 | forward |
|  |  | 4 | CP32_scaffold000086 | 693,282 | reverse |
|  |  | 5 | CP32_scaffold000159 | 289,565 | forward |
|  |  | 6 | CP32_scaffold000022 | 2,747,443 | forward |
|  |  | 7 | CP32_scaffold000021 | 2,780,951 | forward |
|  |  | 8 | CP32_scaffold000020 | 2,794,373 | forward |
| CP4.1LG06 | LG07 | 1 | CP32_scaffold000035 | 2,140,805 | forward |
|  |  | 2 | CP32_scaffold000083 | 758,721 | reverse |
|  |  | 3 | CP32_scaffold000042 | 1,749,822 | forward |
|  |  | 4 | CP32_scaffold000043 | 1,732,427 | reverse |
|  |  | 5 | CP32_scaffold000082 | 760,949 | reverse |
|  |  | 6 | CP32_scaffold000185 | 216,892 | forward |
|  |  | 7 | CP32_scaffold013127 | 333 | forward |
|  |  | 8 | CP32_scaffold000158 | 289,633 | reverse |
|  |  | 9 | CP32_scaffold000036 | 2,105,938 | reverse |
|  |  | 10 | CP32_scaffold000106 | 503,388 | forward |
|  |  | 11 | CP32_scaffold000127 | 408,837 | forward |
| CP4.1LG07 | LG15 | 1 | CP32_scaffold000060 | 1,133,474 | forward |
|  |  | 2 | CP32_scaffold000093 | 615,043 | reverse |
|  |  | 3 | CP32_scaffold000056 | 1,254,317 | forward |
|  |  | 4 | CP32_scaffold000097 | 558,874 | reverse |
|  |  | 5 | CP32_scaffold000069 | 945,996 | reverse |
|  |  | 6 | CP32_scaffold000135 | 366,381 | reverse |
|  |  | 7 | CP32_scaffold000119 | 428,542 | reverse |
|  |  | 8 | CP32_scaffold000133 | 378,800 | undefined |
|  |  | 9 | CP32_scaffold000023 | 2,545,012 | forward |
|  |  | 10 | CP32_scaffold000138 | 352,159 | reverse |
|  |  | 11 | CP32_scaffold000214 | 135,984 | forward |
|  |  | 12 | CP32_scaffold000172 | 241,243 | reverse |
|  |  | 13 | CP32_scaffold000092 | 623,336 | forward |
|  |  | 14 | CP32_scaffold000098 | 555,395 | reverse |
| CP4.1LG08 | LG06 | 1 | CP32_scaffold000003 | 4,875,806 | reverse |
|  |  | 2 | CP32_scaffold000089 | 642,205 | forward |
|  |  | 3 | CP32_scaffold000008 | 3,995,464 | forward |
|  |  | 4 | CP32_scaffold000101 | 542,828 | forward |
| CP4.1LG09 | LG08 | 1 | CP32_scaffold000014 | 3,564,952 | forward |
|  |  | 2 | CP32_scaffold000251 | 101,034 | forward |
|  |  | 3 | CP32_scaffold000061 | 1,116,870 | reverse |
|  |  | 4 | CP32_scaffold000132 | 379,208 | reverse |
|  |  | 5 | CP32_scaffold000245 | 101,953 | forward |
|  |  | 6 | CP32_scaffold000247 | 101,524 | reverse |
|  |  | 7 | CP32_scaffold000145 | 331,494 | undefined |
|  |  | 8 | CP32_scaffold000075 | 863,406 | forward |
|  |  | 9 | CP32_scaffold000072 | 896,126 | forward |
|  |  | 10 | CP32_scaffold000026 | 2,454,755 | forward |
| CP4.1LG10 | LG10 | 1 | CP32_scaffold000009 | 3,958,430 | reverse |
|  |  | 2 | CP32_scaffold000029 | 2,358,218 | reverse |
|  |  | 3 | CP32_scaffold000068 | 950,888 | reverse |
|  |  | 4 | CP32_scaffold000125 | 410,804 | forward |
|  |  | 5 | CP32_scaffold000160 | 285,501 | forward |
|  |  | 6 | CP32_scaffold000057 | 1,171,448 | reverse |
|  |  | 7 | CP32_scaffold000171 | 242,204 | reverse |
|  |  | 8 | CP32_scaffold000114 | 450,599 | reverse |
| CP4.1LG11 | LG13 | 1 | CP32_scaffold000107 | 498,007 | reverse |
|  |  | 2 | CP32_scaffold000017 | 3,213,514 | forward |
|  |  | 3 | CP32_scaffold000108 | 488,883 | forward |
|  |  | 4 | CP32_scaffold000124 | 411,803 | reverse |
|  |  | 5 | CP32_scaffold000141 | 338,006 | forward |
|  |  | 6 | CP32_scaffold000088 | 647,301 | reverse |
|  |  | 7 | CP32_scaffold000113 | 452,942 | reverse |
|  |  | 8 | CP32_scaffold000049 | 1,492,573 | forward |
|  |  | 9 | CP32_scaffold000053 | 1,373,939 | forward |
|  |  | 10 | CP32_scaffold000094 | 602,087 | forward |
|  |  | 11 | CP32_scaffold000153 | 304,914 | forward |
| CP4.1LG12 | LG05 | 1 | CP32_scaffold000012 | 3,815,302 | forward |
|  |  | 2 | CP32_scaffold000121 | 422,947 | reverse |
|  |  | 3 | CP32_scaffold000071 | 899,720 | forward |
|  |  | 4 | CP32_scaffold000018 | 3,164,343 | reverse |
|  |  | 5 | CP32_scaffold000048 | 1,517,882 | reverse |
| CP4.1LG13 | LG16 | 1 | CP32_scaffold000085 | 695,292 | forward |
|  |  | 2 | CP32_scaffold000034 | 2,168,721 | forward |
|  |  | 3 | CP32_scaffold000129 | 385,840 | forward |
|  |  | 4 | CP32_scaffold000225 | 119,458 | reverse |
|  |  | 5 | CP32_scaffold000080 | 770,750 | forward |
|  |  | 6 | CP32_scaffold000028 | 2,396,146 | reverse |
|  |  | 7 | CP32_scaffold000030 | 2,292,851 | reverse |
|  |  | 8 | CP32_scaffold000104 | 518,031 | forward |
| CP4.1LG14 | LG19 | 1 | CP32_scaffold000005 | 4,849,021 | reverse |
|  |  | 2 | CP32_scaffold000147 | 327,288 | undefined |
|  |  | 3 | CP32_scaffold000182 | 222,078 | undefined |
|  |  | 4 | CP32_scaffold000050 | 1,454,989 | forward |
|  |  | 5 | CP32_scaffold000037 | 2,098,557 | forward |
| CP4.1LG15 | LG11 | 1 | CP32_scaffold000015 | 3,441,236 | forward |
|  |  | 2 | CP32_scaffold000131 | 379,828 | forward |
|  |  | 3 | CP32_scaffold000047 | 1,558,130 | forward |
|  |  | 4 | CP32_scaffold000016 | 3,434,250 | forward |
| CP4.1LG16 | LG17 | 1 | CP32_scaffold000067 | 991,265 | reverse |
|  |  | 2 | CP32_scaffold000200 | 168,863 | forward |
|  |  | 3 | CP32_scaffold000164 | 278,388 | undefined |
|  |  | 4 | CP32_scaffold000256 | 97,354 | undefined |
|  |  | 5 | CP32_scaffold000070 | 928,547 | forward |
|  |  | 6 | CP32_scaffold000206 | 156,815 | undefined |
|  |  | 7 | CP32_scaffold000152 | 310,330 | forward |
|  |  | 8 | CP32_scaffold000100 | 550,348 | forward |
|  |  | 9 | CP32_scaffold000142 | 337,580 | forward |
|  |  | 10 | CP32_scaffold000004 | 4,863,444 | forward |
| CP4.1LG17 | LG12 | 1 | CP32_scaffold000103 | 531,021 | reverse |
|  |  | 2 | CP32_scaffold000065 | 997,141 | forward |
|  |  | 3 | CP32_scaffold000074 | 869,430 | reverse |
|  |  | 4 | CP32_scaffold000096 | 589,541 | forward |
|  |  | 5 | CP32_scaffold000120 | 423,438 | reverse |
|  |  | 6 | CP32_scaffold000186 | 215,517 | forward |
|  |  | 7 | CP32_scaffold000058 | 1,167,845 | forward |
|  |  | 8 | CP32_scaffold000031 | 2,250,994 | reverse |
|  |  | 9 | CP32_scaffold000045 | 1,627,577 | forward |
| CP4.1LG18 | LG14 | 1 | CP32_scaffold000073 | 884,058 | forward |
|  |  | 2 | CP32_scaffold000095 | 591,145 | forward |
|  |  | 3 | CP32_scaffold000116 | 432,341 | forward |
|  |  | 4 | CP32_scaffold000157 | 293,129 | forward |
|  |  | 5 | CP32_scaffold000112 | 453,344 | forward |
|  |  | 6 | CP32_scaffold000039 | 1,853,076 | forward |
|  |  | 7 | CP32_scaffold000011 | 3,820,361 | forward |
| CP4.1LG19 | LG09 | 1 | CP32_scaffold000054 | 1,345,344 | forward |
|  |  | 2 | CP32_scaffold000064 | 1,081,996 | forward |
|  |  | 3 | CP32_scaffold000161 | 281,296 | forward |
|  |  | 4 | CP32_scaffold000197 | 183,073 | reverse |
|  |  | 5 | CP32_scaffold000146 | 330,575 | forward |
|  |  | 6 | CP32_scaffold000077 | 836,762 | reverse |
|  |  | 7 | CP32_scaffold000130 | 382,378 | reverse |
|  |  | 8 | CP32_scaffold000013 | 3,798,258 | reverse |
| CP4.1LG20 | LG21 | 1 | CP32_scaffold000007 | 4,346,540 | forward |
|  |  | 2 | CP32_scaffold000148 | 327,036 | reverse |
|  |  | 3 | CP32_scaffold000115 | 449,779 | forward |
|  |  | 4 | CP32_scaffold000110 | 469,294 | forward |
|  |  | 5 | CP32_scaffold000151 | 313,452 | forward |
|  |  | 6 | CP32_scaffold000081 | 769,043 | reverse |
|  |  | 7 | CP32_scaffold000052 | 1,439,660 | reverse |
