# Supplementary material for: De novo assembly of the zucchini genome reveals a whole‐genome duplication associated with the origin of the Cucurbita genus
Source: Plant Biotechnol J. 2017 Dec 4;16(6):1161–71. doi: 10.1111/pbi.12860 (PMC5978595; doi:10.1111/pbi.12860)
Supplement: Supplementary file 13 — Table S5 Summary of repetitive elements found in Cucurbita pepo, Cucumis melo, Cucumis sativus and Citrullus lanatus. All results are expressed in bp. [file PBI-16-1161-s008.docx]

Supplementary Table 5. Summary of repetitive elements found in *Cucurbita pepo, Cucumis melo, Cucumis sativus* and *Citrullus lanatus.* All results are expressed in bp.

| **Size (bp)** | ***Cucurbita pepo*** | ***Cucumis melo*** | ***Citrullus lanatus*** | ***Cucumis sativus* Chinese long** | ***Cucumis***  ***sativus* PI183967** |
| --- | --- | --- | --- | --- | --- |
| Genome size | 289504453 | 406928820 | 355247419 | 197271687 | 204803225 |
| Genome size without Ns | 247816929 | 336097957 | 321405453 | 193700889 | 200988521 |
| Repetitive | 93650597 | 190225685 | 186381889 | 55566601 | 63676019 |
| Repetitive (no overlapping) | 85581680 | 179982107 | 173126881 | 52923711 | 60250642 |
| over_same | 4361793 | 5007047 | 5992548 | 1199958 | 1697636 |
| over_diff | 3707124 | 5236531 | 7262460 | 1442932 | 1727741 |
| DNA |  | 193677 | 283426 | 54557 |  |
| DNA/CMC-EnSpm | 88523 | 11363266 | 2589519 | 1551581 | 1955210 |
| DNA/Crypton-C |  |  | 7193 |  |  |
| DNA/En-Spm |  |  | 139908 |  |  |
| DNA/Ginger |  |  | 121896 | 134666 |  |
| DNA/IS3EU |  |  |  |  | 82374 |
| DNA/Kolobok-T2 | 27949 |  |  |  | 58226 |
| DNA/MULE-MuDR | 145882 | 4697101 | 1633726 | 1195740 | 1003127 |
| DNA/Maverick |  |  | 192173 |  |  |
| DNA/MuDR |  | 929167 | 122428 | 136004 | 148317 |
| DNA/MuLE-MuDR |  | 6548932 | 4029767 | 890154 | 996088 |
| DNA/PIF-Harbinger | 12213 | 3090436 | 1290961 | 550468 | 343060 |
| DNA/TcMar-Mariner |  |  | 3360 |  |  |
| DNA/Zisupton |  |  |  | 25310 |  |
| DNA/hAT-Ac | 1246686 | 728666 | 1709109 | 264199 | 479104 |
| DNA/hAT-Charlie |  | 148193 | 38283 | 60790 |  |
| DNA/hAT-Tag1 | 159459 | 383200 | 478025 | 345958 | 361065 |
| DNA/hAT-Tip100 |  | 379594 | 83286 | 367630 | 83315 |
| DNA/hAT-hATm |  |  | 2825369 |  |  |
| LINE/CR1 |  |  |  | 36758 | 21279 |
| LINE/CRE | 169102 |  | 31891 | 20041 |  |
| LINE/I-Jockey |  | 301 |  |  |  |
| LINE/L1 | 2207438 | 5388043 | 5944566 | 2957971 | 3237632 |
| LINE/L1-DRE |  |  | 217246 |  | 86388 |
| LINE/L1-Tx1 | 194920 | 201316 | 224728 | 23383 |  |
| LINE/L2 |  | 24417 | 950717 | 70984 |  |
| LINE/RTE-BovB | 143 |  | 185123 |  | 104075 |
| LINE/Tad1 | 68219 |  |  |  | 117729 |
| LTR | 65446 | 200156 | 263981 | 334749 |  |
| LTR/Cassandra | 1310467 | 387478 | 346831 | 236843 | 41046 |
| LTR/Caulimovirus | 719038 | 1100094 | 225529 | 122829 | 55250 |
| LTR/Copia | 6667202 | 39238863 | 33496488 | 9503582 | 10759234 |
| LTR/DIRS | 150502 |  |  |  |  |
| LTR/ERV1 |  | 189116 |  |  | 12581 |
| LTR/ERVK | 30609 |  |  |  |  |
| LTR/ERVL |  |  | 136788 |  |  |
| LTR/Gypsy | 8160792 | 43716718 | 31453573 | 5844651 | 7988269 |
| LTR/Pao |  | 12326 |  |  | 32675 |
| RC/Helitron | 181502 | 632496 | 114736 | 71766 | 48395 |
| Retroposon |  |  |  | 73613 |  |
| SINE/Alu |  |  |  |  | 48743 |
| SINE/B2 | 11862 |  | 11186 |  |  |
| SINE/ID | 175670 |  |  |  |  |
| SINE/tRNA | 200088 | 333231 | 686539 | 255170 | 246352 |
| SINE/tRNA-R2 | 23160 |  |  |  |  |
| SINE/tRNA-RTE | 76347 |  |  |  |  |
| SINE? | 1376290 | 2458 | 20168 |  | 41669 |
| Satellite | 399460 | 232131 |  | 13933 | 6340 |
| Satellite/Y-chromosome | 31503 |  |  |  |  |
| Satellite/centr |  |  |  |  | 63047 |
| Simple repeats | 8092785 | 5360259 | 5874319 | 3676307 | 4190723 |
| Low complexity regions | 1229916 | 1395906 | 1646367 | 1175901 | 1160849 |
| rRNA | 468015 | 176725 | 41472 | 50262 | 109384 |
| snRNA | 29907 | 7471 | 6485 | 6750 | 4777 |
| Unknown/undefined | 59929502 | 63163948 | 88954727 | 25514051 | 29789696 |
