# Supplementary material for: De novo assembly of the zucchini genome reveals a whole‐genome duplication associated with the origin of the Cucurbita genus
Source: Plant Biotechnol J. 2017 Dec 4;16(6):1161–71. doi: 10.1111/pbi.12860 (PMC5978595; doi:10.1111/pbi.12860)
Supplement: Supplementary file 14 — Table S6 Gene family (orthogroups and paralogs in OrthoMCL) identification. [file PBI-16-1161-s009.docx]

Supplementary Table 6. Gene family (orthogroups and paralogs in OrthoMCL) identification.

| **Species** | **# proteins** | **# assigned to a gene family (several species)** | **% assigned** | **# assigned to a gene family (species exclusive)** | **% assigned to a gene family (species exclusive)** | **% of proteins assigned** |
| --- | --- | --- | --- | --- | --- | --- |
| *Cucurbita pepo* | 27,870 | 25,433 | 91.23 | 291 | 1.04 | 95.26 |
| *Citrullus lanatus* | 23,440 | 18,798 | 80.20 | 2,601 | 11.10 | 94.16 |
| *Cucumis melo* | 27,427 | 19,974 | 72.83 | 3,570 | 13.02 | 88.22 |
| *Cucumis sativus* Chinese Long | 23,248 | 19,111 | 82.20 | 394 | 1.69 | 86.94 |
| *Cucumis sativus* PI183967 | 22,790 | 19,360 | 84.95 | 370 | 1.62 | 89.48 |
